# Impact of Acute and Chronic Psychosocial Stress on Vascular Inflammation

**DOI:** 10.1089/ars.2021.0153

**Published:** 2021-12-01

**Authors:** Julia Hinterdobler, Heribert Schunkert, Thorsten Kessler, Hendrik B. Sager

**Affiliations:** ^1^ Department of Cardiology, German Heart Centre Munich, Technical University Munich, Munich, Germany.; ^2^ DZHK (German Centre for Cardiovascular Research), partner site Munich Heart Alliance, Munich, Germany.

**Keywords:** psychosocial stress, vascular inflammation, risk factors for atherosclerosis, neuroimmune interactions

## Abstract

***Significance:*** Atherosclerosis and its complications, such as acute coronary syndromes, are the leading causes of death worldwide. A wide range of inflammatory processes substantially contribute to the initiation and progression of cardiovascular disease (CVD). In addition, epidemiological studies strongly associate both chronic stress and acute psychosocial stress with the occurrence of CVDs.

***Recent Advances:*** Extensive research during recent decades has not only identified major pathways in cardiovascular inflammation but also revealed a link between psychosocial factors and the immune system in the context of atherosclerosis. Both chronic and acute psychosocial stress drive systemic inflammation *via* neuroimmune interactions and promote atherosclerosis progression.

***Critical Issues:*** The associations human epidemiological studies found between psychosocial stress and cardiovascular inflammation have been substantiated by additional experimental studies in mice and humans. However, we do not yet fully understand the mechanisms through which psychosocial stress drives cardiovascular inflammation; consequently, specific treatment, although urgently needed, is lacking.

***Future Directions:*** Psychosocial factors are increasingly acknowledged as risk factors for CVD and are currently treated *via* behavioral interventions. Additional mechanistic insights might provide novel pharmacological treatment options to reduce stress-related morbidity and mortality. *Antioxid. Redox Signal.* 35, 1531–1550.

## Introduction

Atherosclerosis and its complications, such as acute myocardial infarction (MI) and stroke, are the leading causes of death worldwide (136). A chronic disease of the vessel wall, atherosclerosis may ultimately lead to lumen narrowing, partial or complete obstruction, and reduced blood flow to downstream organs (91). Despite considerable progress in treating atherosclerosis, the prevalence of its complications almost doubled in the past three decades (132, 136).

Extensive research into the underlying mechanisms has identified that inflammatory—along with metabolic—components significantly contribute to the etiology of atherosclerosis (90). In this context, various studies have demonstrated that classical and nonclassical cardiovascular risk factors are involved in the pathology of atherosclerosis *via* the modulation of inflammatory processes (146). Psychosocial stress in particular is a cardiovascular risk factor that is strongly linked to cardiovascular disease (CVD) (75). The complex interplay of the nervous, hormonal, metabolic, and immune systems during stress responses harbors great potential for interventions in the ways psychosocial stress impacts cardiovascular inflammation. In this review, we discuss recent scientific discoveries and developments that investigate the effects of acute and chronic psychosocial stress on cardiovascular inflammation.

## The Role of Inflammation in the Pathogenesis of Atherosclerosis

Atherosclerosis is the underlying pathology for a variety of cardiovascular complications and has long been considered a largely metabolic disease, caused by high plasma cholesterol levels and passive lipid accumulation inside the vessel wall (141). However, in recent decades, a great number of experimental, epidemiological, and lately also clinical studies have convincingly shown that inflammation plays an important role in the etiology of atherosclerosis (1, 133, 163, 176). This is reinforced by the fact that the incidence of CVD remains high, even after efficient lipid-lowering treatment became widely used (77, 134).

Various experimental studies have dissected the mechanisms that underlie atherosclerosis initiation and progression. As a first step, in a setting with high plasma low-density lipoprotein (LDL) levels and additional proinflammatory stimuli, endothelial dysfunction in atherosclerosis-prone regions with disturbed flow favors the uptake of cholesterol-loaded LDL. This promotes the upregulation of adhesion molecules on endothelial cells and the subsequent recruitment of inflammatory leukocytes (93). Among these recruited cells, specifically dedicated blood monocyte-derived macrophages clear both nonmodified and oxidized cholesterol particles and then become foam cells (89).

If initial clearance fails, inflammation persists and is further promoted by a wide range of leukocytes that release proinflammatory mediators such as interleukin (IL) 1ꞵ, tumor necrosis factor alpha (TNFα), and various other cytokines and chemokines (1, 190). To separate the plaque content from the lumen, smooth muscle cells (SMCs) are recruited from the tunica media to the tunica intima and produce an extracellular matrix that forms a fibrous cap and stabilizes the atheromatous plaque (161).

**FIG. 1.**
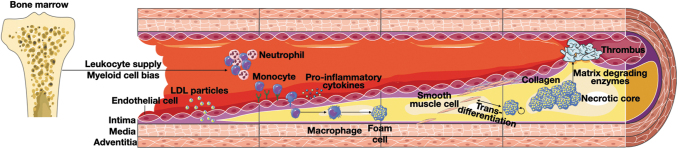
**Cellular processes involved in atherosclerosis development.** Atherosclerotic lesion development is initiated by LDL particle uptake from the blood into the intimal regions of the vessel wall. The activated endothelium in these regions upregulates adhesion molecule and cytokine expression, leading to blood leukocyte recruitment. This is further promoted by increased inflammatory leukocyte production in the bone marrow caused by systemic inflammation, hyperlipidemia, and hyperglycemia. Once inside the vessel wall, recruited monocytes differentiate into macrophages and develop into foam cells when they engulf excess amounts of cholesterol. SMCs transmigrate from the vascular media to the intima and produce collagen to stabilize the fibrous cap that separates the plaque content from the lumen. Upon lesion progression, foam cells further accumulate and form a necrotic core. Eventually, persistent inflammatory processes culminate in fibrous cap breakdown through, for example, matrix-degrading enzymes, and cause thrombotic complications. LDL, low-density lipoprotein; SMC, smooth muscle cell.

In general, lesions with a thick fibrous cap, low inflammatory cell activity, and small necrotic cores (accumulation of lipids and apoptotic macrophages inside the atheroma) are considered stable plaques (152). However, these plaques can transition into unstable/vulnerable plaques. Plaque inflammatory leukocytes can promote extracellular matrix degradation and SMC death, which can ultimately lead to plaque rupture or erosion with atherothrombosis that causes cardiovascular complications such as MI or stroke (92). Figure 1 illustrates the cellular processes that lead to the initiation and progression of atherosclerotic lesions.

### The role of neutrophils in vascular inflammation

The innate immune system has long been considered the first line of defense against infections, as innate immune system effector cells, such as neutrophils, are rapidly recruited to sites of acute inflammation (150). However, innate immune cells are now widely understood as contributors to chronic sterile inflammation that occurs in atherosclerosis (27, 53). Neutrophils contribute to all stages of the disease: initiation, progression, and atherosclerotic lesion rupture (158).

As described above, lipid accumulation in the vessel wall causes endothelial dysfunction and immune cell recruitment. A growing body of evidence indicates that neutrophils are among the first cells recruited to early atherosclerotic lesions and contribute significantly to the subsequent recruitment of, for example, inflammatory monocytes (141, 158). High cholesterol levels affect not only the recruitment of neutrophils to atherosclerotic lesions but also their production in the bone marrow (150). Experimental studies could prove that hypercholesterolemia induces the proliferation of hematopoietic stem cells with a specific bias toward the myeloid (neutrophils, monocytes, and macrophages) lineage, resulting in blood neutrophilia and monocytosis (21, 29, 189).

Upon activation, neutrophils promote lesion progression through various mechanisms, including macrophage activation and the release of neutrophil extracellular traps, proinflammatory web-like structures consisting of DNA and proteins (45, 151). Ultimately, neutrophils contribute to plaque destabilization by releasing a wide spectrum of proinflammatory mediators and matrix-degrading enzymes (41, 101).

In line with their mechanistic function in the pathology of atherosclerosis, high levels of neutrophils in plasma and inside atherosclerotic lesions have been associated with an increased risk for cardiovascular events (63, 94). Indeed, a recent analysis of five large randomized clinical trials showed that the neutrophil–lymphocyte ratio is a readily available inflammatory biomarker that predicts cardiovascular outcomes (2). Moreover, the neutrophil–lymphocyte ratio is modulated by canakinumab—a monoclonal antibody against proinflammatory IL-1ꞵ—and may therefore help guide anti-inflammatory treatment (2).

### The role of monocytes and macrophages in vascular inflammation

Similar to neutrophils, inflammatory monocytes are recruited from blood to sites of inflammation (141). In early atherosclerosis, the inflamed endothelium expresses adhesion molecules and chemokines that promote monocyte recruitment (129). Activated endothelial cells raise the expression of intercellular adhesion molecule 1 (ICAM-1), vascular adhesion molecule 1 (VCAM-1) and selectins, among others, and secrete proinflammatory and leukocyte-attracting chemokines such as CC-chemokine ligand (CCL) 2 (60). As mentioned above, prior neutrophil recruitment is thought to further enhance inflammation and consequently promote monocyte recruitment.

Once inside the atherosclerotic lesion, most inflammatory monocytes differentiate into macrophages dedicated to ingesting cholesterol particles (107). The resulting lipid-loaded macrophages, termed foam cells, further fuel inflammation by, for example, secreting IL-1ꞵ (30). Of note, recent studies have demonstrated that macrophage accumulation in atherosclerosis is also driven by local proliferation and SMC transdifferentiation into macrophage-like cells (54, 92, 135).

In advanced atherosclerotic lesions, macrophages contribute to the development of unstable plaques as prolonged inflammation and disturbed efferocytosis (phagocytic clearance of apoptotic cells) promote macrophage death and necrotic core expansion (141). In addition, activated macrophages produce different kinds of proteinases, such as matrix metalloproteinases, that lead to fibrous cap breakdown (90, 107). Although macrophages are generally considered the main drivers of inflammation in atherosclerosis, accumulating evidence has demonstrated the heterogeneity of lesional macrophages, highlighted the importance of macrophages in atherosclerosis regression, and thus propagated a more nuanced view of the role of macrophages in atherosclerosis (7, 38, 170, 183).

As mentioned above, both hyperlipidemia and hyperglycemia contribute to high blood monocyte levels in atherosclerosis by increasing their production in the bone marrow. In addition, release from the bone marrow is promoted by circulating chemokines such as CCL2, CCL7, and CXC-chemokine ligand 1 (CXCL1) (28, 166). Similar to neutrophils, high numbers of inflammatory blood monocytes are strongly associated with CVD risk (8, 49).

### The role of lymphocytes in vascular inflammation

While the role of innate immune cells in atherosclerosis is widely acknowledged, adaptive immune responses also contribute to all stages of atherosclerosis (56). Lymphocytes, namely T and B cells, are the cellular key players of the adaptive immune system with their reactions being highly specific and long-lasting (72). In atherosclerosis initiation, myeloid infiltration, as a response to LDL accumulation, is accompanied by the recruitment of both T and B cells (44). Some of these cells harbor a specific receptor to recognize the core component of LDL, apolipoprotein B, which has led to the notion that atherosclerosis is an inflammatory disease that involves autoimmune processes (184).

Although both T and B cells can be found in atherosclerotic plaques, T cells are more prominent throughout all stages of the disease and constitute approximately one-fourth of all plaque leukocytes (72, 184). T lymphocytes have been found to play controversial roles in atherosclerosis progression. Both the predominant cluster of differentiation (CD) 4^+^ effector T cells and the cytotoxic CD8^+^ T cells have been shown to be proatherogenic, while some CD4^+^ regulatory T cells exert highly atheroprotective functions (56, 194). T cell polarization inside the plaque occurs as a result of the interaction with antigen-presenting cells (184). Here, costimulatory molecules on the interacting cells and cytokines released by the antigen-presenting cell decide on the fate of the naive T cell, which results in the activation into either a pro- or antiatherogenic phenotype. Based on their polarization, mature T cells exert their pro- or anti-inflammatory functions by effecting other T cells, B cells, and tissue-resident cells (184).

Classically, B cells can be distinguished into B1 cells that are part of the innate immune system, and B2 cells that can differentiate into plasma cells and secrete immunoglobulin G (IgG) antibodies (184). Both types are found in atherosclerotic plaques although less frequent than T cells. While B1 cells are attributed to be exclusively atheroprotective, B2 cells seem to exert both pro- and antiatherogenic responses inside plaques (3, 17, 82)

Taken together, the adaptive immune system is involved in pro- and anti-inflammatory processes in atherosclerosis. Current models suggest a general transition from antiatherogenic to proatherogenic mechanisms during disease progression although it is still unclear if this switch is a cause or a consequence (184). Although research has discovered multiple roles of the adaptive immune system in CVD, experimental studies on the risk factors of atherosclerosis have predominantly focused on the roles of the innate system until now.

## Risk Factors of Atherosclerosis

### Classical risk factors

Certain risk factors and cardiovascular inflammation are connected throughout the pathophysiological processes leading to the development of atherosclerosis. Some classical risk factors are largely nonmodifiable. Being male, for example, is one such classical risk factor for CVD risk, and recent studies link at least part of its association with increased vascular inflammation in both animals and humans (97).

Aging is yet another independent risk factor for CVD; biological aging is associated with various inflammatory processes such as increased oxidative stress or more circulating inflammatory cytokines (167, 177). Clonal hematopoiesis, the process in which increasing numbers of white blood cells derive from one single clone due to specific somatic mutations in hematopoietic stem cells (64), has recently been identified as a pathophysiological mechanism that links aging with atherosclerosis (65). In atherosclerotic mice, clonal hematopoiesis resulted in a stronger activation of inflammatory pathways and thus accelerated atherosclerosis progression (43).

Hereditary components also contribute to CVD risk, and a multitude of genetic loci have been associated with CVD in large-cohort, genome-wide association studies (17a, 55). Interestingly, many of these loci are associated with genes that are part of inflammatory pathways (100). Additional to an inherent genetic component in atherosclerosis itself, certain classical risk factors such as high cholesterol levels, arterial hypertension, and diabetes also harbor some degree of genetic predisposition (15, 46a, 95).

As outlined above, high cholesterol levels contribute to the initiation and progression of atherosclerosis by acting as an important proinflammatory stimulus. Of note, obesity and unhealthy diet, which are also considered classical risk factors for atherosclerosis (35, 121, 178), strongly promote high cholesterol levels (19). Arterial hypertension is another classical risk factor and acts on the integrity of the endothelial cell layer (98). Ongoing research is intensively investigating the link between atherosclerosis and diabetes, with the general proinflammatory milieu in diabetic patients most likely being a major player in the development of atherosclerosis (70, 123).

Smoking is yet another classical risk factor for cardiovascular inflammation and promotes atherosclerosis *via* both local and systemic proinflammatory effects, for example, by circulating toxic compounds and increasing hematopoietic stem cell proliferation (111, 149, 154). Elevated hematopoiesis contributes to atherosclerosis progression by enhancing inflammatory leukocyte supply (117, 146).

### Nonclassical risk factors

**FIG. 2.**
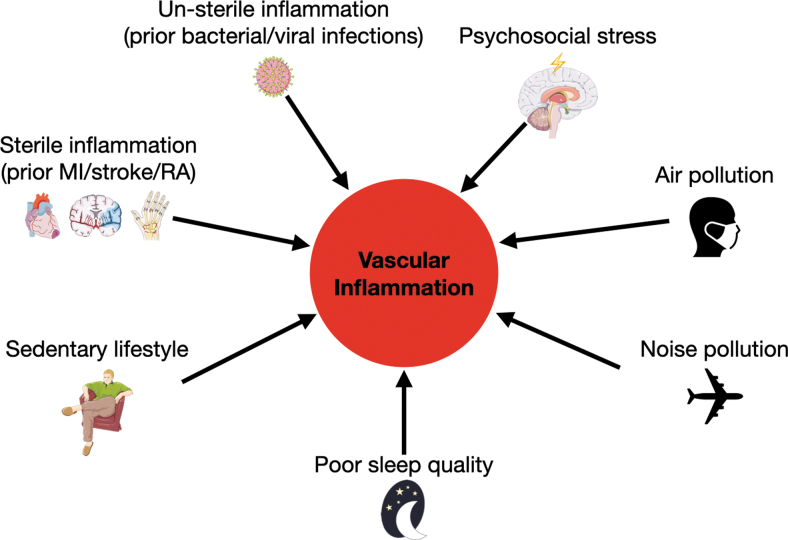
**Nonclassical risk factors contribute to the development of cardiovascular inflammation.** Apart from classical risk factors such as sex, age, genetic predisposition, and blood cholesterol levels, recent research has identified further factors that promote cardiovascular inflammation. Several publications have shown that prior infections, coexisting pathologies, sedentary lifestyle, poor sleep quality, noise and air pollution, and psychosocial stress can all contribute to the development of atherosclerosis. MI, myocardial infarction; RA, rheumatoid arthritis.

It has become increasingly clear that apart from classical risk factors, additional lifestyle factors and other coexisting pathologies influence CVD (Fig. 2). Many of these risk factors have only recently been identified and are thus referred to as nonclassical or nontraditional risk factors. Both viral and bacterial respiratory infections create a higher risk for MI not only during the acute infection but also in the postinfection phase (81, 114). Mechanistically, the acute increase in CVD events may be caused by elevated procoagulant activity, while long-term effects may exacerbate vascular inflammation and hence promote progression of atherosclerosis (140). Moreover, sterile inflammatory pathologies such as prior MI, stroke, or rheumatoid arthritis are linked to increased cardiovascular risk (31, 153). Mechanistically, both nonsterile and sterile inflammations partly exert their effects on cardiovascular inflammation by raising the proinflammatory leukocyte supply through increased production in the bone marrow (31, 32, 140), a mechanism shared by other nonclassical risk factors such as sedentary lifestyle and sleep deprivation (42, 102, 146).

Regular exercise reduces cardiovascular risk through metabolic and antihypertensive effects (86), and a recent study in mice demonstrated that physical activity (simulated by voluntary wheel running) also mitigates cardiovascular inflammation (42). The researchers showed that regular exercise reduces leptin levels and blood leukocyte numbers in mice and humans. In mice, low leptin levels induce bone marrow niche quiescence and retention factors, which limit proinflammatory leukocyte supply. As a consequence, cardiovascular inflammation and atherosclerotic plaque size are curtailed in exercising mice.

A similar picture is emerging with regard to poor sleep quality or sleep deprivation. While epidemiological studies had already linked insufficient sleep to higher cardiovascular risk (13, 162, 165), a recent study in mice uncovered a direct link between sleep and cardiovascular inflammation (102). Here again, the effect on atherosclerosis is mediated *via* increased proinflammatory leukocyte production in the bone marrow: sleep fragmentation causes the hypothalamus to generate less hypocretin, a circulating hormone that normally restricts myeloid cell production in the bone marrow.

Furthermore, the impact of air, light, and noise pollution on CVD health has long been underestimated (87, 112). While air pollution can directly act on inflammatory pathways, noise pollution likely increases CVD risk by modulating other lifestyle risk factors such as sleep and psychosocial stress (112). In detail, air pollution has been shown to influence a wide range of inflammatory processes, including blood leukocyte levels, leukocyte activation, circulating cytokine levels, and endothelial dysfunction (110, 113), which can all act on cardiovascular inflammation. Indeed, air pollution promoted cardiovascular inflammation in mouse models of atherosclerosis (105). Still, it is not yet clear if the effects of air pollution occur primarily through direct action of circulating fine particulate matter (2.5 μm in diameter and less), systemic neuroendocrine activation, and/or systemic inflammation caused by lung injury. In contrast, noise pollution presumably exerts indirect effects by activating physiological stress responses, which are further outlined below. Stress perception in this context may be both conscious (during daytime) and subconscious (during sleep) (112). Data from animal experiments indicate that noise pollution may act on cardiovascular inflammation *via* various mechanisms, including circulating cytokines, endothelial dysfunction, and neutrophil infiltration into atherosclerotic plaques (79, 109).

Over the past years, the term “exposome” has been established to summarize the cumulative effect of lifelong, conscious or unconscious, environmental risk factor exposure (23, 171). International projects/consortia such as “ENNAH” (European Network on Noise and Health), “ELAPSE” (Effects of Low Level Air Pollution: A Study in Europe), and EXPOsOMICS seek to reveal how a combination of different environmental risk factors influence each other and how they affect human health including CVD (20, 39, 85, 173). The exposome concept includes air pollution and noise pollution, and also other factors such as chemical pollution, diet, sleep disruption, social isolation, and work stress (23).

Psychosocial stress, both chronic and acute, is yet another independent risk factor for atherosclerosis that in the past few years has been closely investigated in the context of cardiovascular inflammation. This review aims to summarize the latest advances in this specific research area.

#### Psychosocial stress in vascular inflammation and CVD

Stress has been defined as a constellation of events comprising a stimulus (stressor) that precipitates a reaction in the brain (stress perception) and activates physiologic fight-or-flight systems in the body (175). Psychosocial stressors can be classified as chronic or acute, based on exposure severity and duration (26). Determining strict discrimination criteria, however, is hardly possible, and parameters vary widely between scientific publications. In general, chronic stress is considered exposure to low or moderate and consistent or repetitive stressors such as caring responsibilities, job strain, job insecurity, social isolation, lack of social support, financial stress, marital unhappiness, long-term depression, hopelessness, loneliness, and type A or D personality (75). In contrast, acute stress refers to short-term (minutes up to days) and severe stressors such as anger outburst, emotional upset, anxiety, sadness, grief, bereavement, natural disasters, acts of war and terrorism, major sporting events, and work stress (high-pressure deadline at work) (106).

Naturally, acute stress can also transition into chronic stress, and perceived severity strongly depends on individual stress susceptibility (33). The distinction between distress (negative stress) and eustress (positive stress) is yet another way to classify psychosocial stressors (12), and individual perception plays an even bigger role in this differentiation. For simplicity, this review only summarizes current literature on the effects of distress on cardiovascular inflammation.

**FIG. 3.**
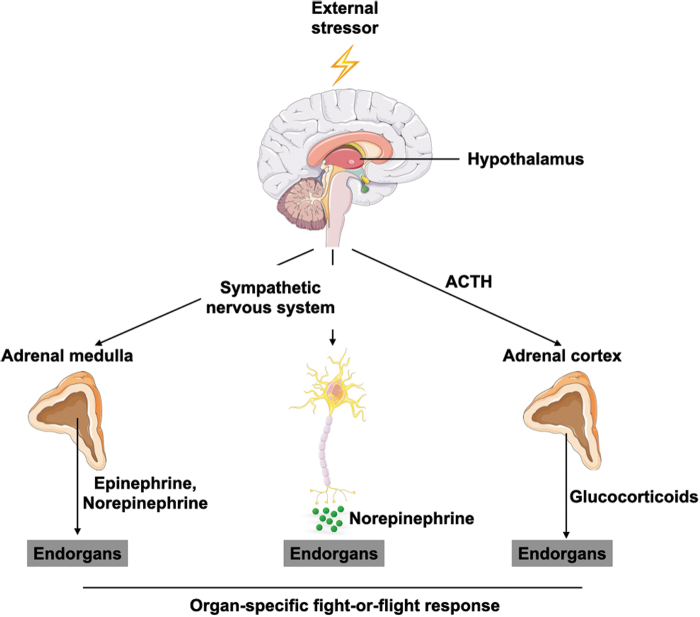
**Stress perception activates distinct physiological stress responses.** Once the brain perceives an external stressor, the body attempts to restore homeostasis by activating systemic stress responses. The HPA axis results in the release of glucocorticoids from the adrenal cortex, while sympathetic nervous system activation causes systemic (*via* the adrenal medulla) and local (*via* sympathetic nerve endings) release of catecholamines. Altogether, these act on respective end organs to prepare the body for a fight-or-flight situation. ACTH, adrenocorticotropic hormone; HPA, hypothalamus/pituitary/adrenal.

Exposure to an external stressor promotes the activation of a physiological stress response. From an evolutionary perspective, this reaction is essential to ensure survival in so-called fight-or-flight situations (116). To trigger a fight-or-flight response, the respective stimulus needs to be perceived by the brain, where subsequent neuronal networks activate three major pathways in the body (Fig. 3) (83).

One pathway is the hypothalamus/pituitary/adrenal (HPA) axis. Here, the hypothalamus releases the corticotropin releasing hormone (CRH), which leads the pituitary gland to secrete the adrenocorticotropic hormone (ACTH) into the blood stream. In the adrenal cortex, ACTH leads to systemic release of glucocorticoid hormones such as cortisol. The other two pathways exert their functions *via* the sympathetic nervous system (SNS). The sympatho-adrenomedullary (SAM) axis involves sympathetic innervation of the adrenal medulla, which releases systemic catecholamines (epinephrine and norepinephrine) in response to stimulation. SNS activation through stress perception can also directly activate dedicated end organs *via* sympathetic nerve endings locally releasing norepinephrine.

The purpose of this physiological response is to prepare the body to react to threats and endure potential injuries (116). Body functions necessary for bolstering survival, such as blood perfusion of skeletal muscles, energy mobilization, ventilation of the lungs and heart rate, are increased while supply to, for example, the digestive and reproductive systems, is curtailed (125).

Neuroimmunology is a rapidly developing research area studying interactions between the nervous and immune systems. Both systems are responsible for maintaining homeostasis despite adverse environmental stimuli, and it is widely acknowledged that they operate together to exert appropriate adaptations to potential threats (142). Stress situations promote neuroimmune interactions, but they also occur constantly under steady-state conditions during which they control, for example, circadian leukocyte trafficking (62, 145). In this context, the SNS seems to play a specific pivotal role in rhythmic leukocyte recruitment (66).

Interactions between the nervous and immune systems are bidirectional, but signaling from the nervous to the immune system seems to be especially critical with regard to the negative effects of stress on human health (47). Despite contributing to a low-grade inflammatory milieu in the body, psychosocial stress is thought to compromise the body's ability to mount a proper immune reaction in response to infections. Accordingly, stimulating adrenergic signaling over 7 days resulted in reduced host resistance to viral infections in a study investigating how adrenergic signaling affects the innate immune response in a mouse model (180). Furthermore, a recent publication showed that acute stress leads the adipose tissue to release more IL-6, which is needed to ensure glucose supply in a fight-or-flight situation but would have detrimental effects regarding concomitant bacterial infection (128).

Other studies, by contrast, suggest that acute psychosocial stress has a short-term immune-enhancing effect, for example, by mobilizing immune cells from the bone marrow or redistributing leukocytes to sites of inflammation (25, 175). Overall, the effect of psychosocial stress on the immune system may depend on the combination of the stress duration and the specific type of environmental challenge in which it occurs (62).

In the context of vascular inflammation and atherosclerosis, we have just begun to explore neuroimmune interactions. Nervous system activity is critical to the regulation of stem cell proliferation in hematopoietic organs and the release of mature leukocytes (18, 71, 124). Of note, sympathetic neuronal activation promotes the proliferation of splenic hematopoietic progenitor cells with a concomitant myeloid cell bias (169). Recent publications have also demonstrated that the nervous system directly affects immune processes in the vessel wall (18). Depleting the SNS, for instance, reversed adhesion molecule upregulation by aortic endothelial cells in a mouse model of MI (139).

**FIG. 4.**
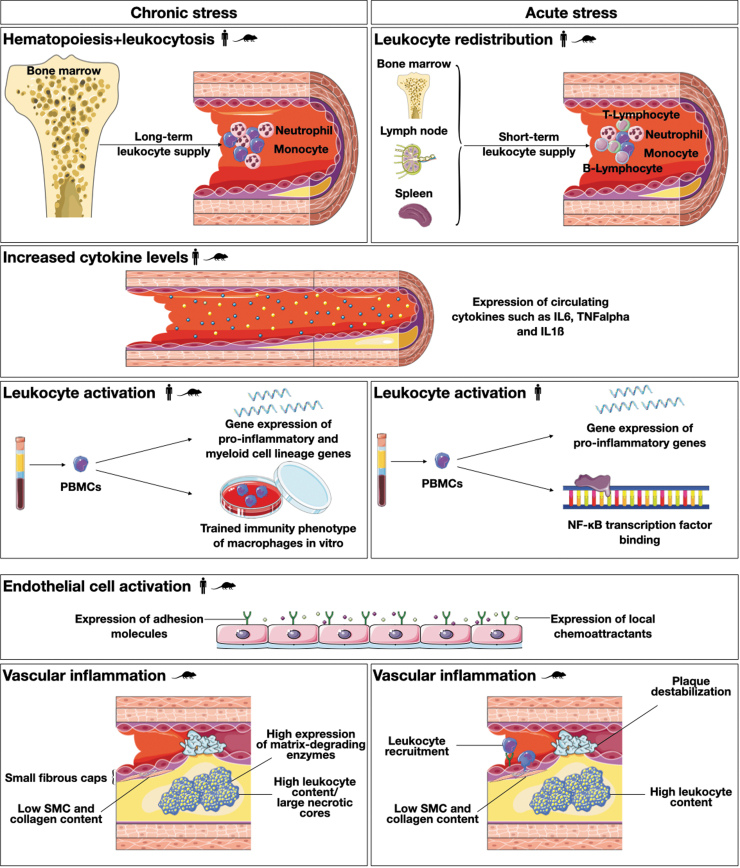
**The role of psychosocial stress in cardiovascular inflammation.** Both chronic and acute psychosocial stress act as major players in the inflammatory cascades that lead to the initiation and progression of atherosclerosis. This figure summarizes current evidence regarding how chronic and acute psychosocial stress affect leukocyte distribution, systemic proinflammatory signaling, leukocyte and endothelial cell activation, and inflammatory processes inside the vessel wall. 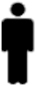 = Evidence from human experimental data, 
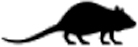
 = evidence from animal experimental data. PBMC, peripheral blood mononuclear cell; SMC, smooth muscle cell.

It is only logical that neuroimmune interactions are also involved in linking psychosocial stress and cardiovascular inflammation. Experimental studies on psychosocial stress have identified three major areas with potential influence on CVD. First, the physiological stress response strongly influences hemodynamic parameters such as cardiac output, blood pressure, and heart rate (138). Second, activation of the different stress axes can also affect hemostatic parameters such as platelet activation (67, 78). In the following sections, this review seeks to summarize experimental evidence for the third area: the inflammatory contribution of psychosocial stress to CVD risk (Fig. 4).

##### Chronic psychosocial stress

Results from the INTERHEART study provide a large body of evidence for the association between psychosocial stress and CVD (188). This large case/control study in 52 countries quantified the relationships between major cardiovascular risk factors, including long-term psychosocial factors, and the occurrence of MI. In this study, the prevalence of psychosocial stress is associated with an odds ratio of 2.67 for MI. In contrast to the INTERHEART study, which analyzed psychosocial stressors in a summarized category, other large-scale studies investigated the links between CVD and isolated long-term psychosocial stressors, such as job strain, childhood abuse, and social isolation. Meta-analyses of corresponding studies reported odds ratios of up to 1.5 for associations between psychosocial stressors and cardiovascular endpoints (75, 174). Results from the Whitehall II study highlighted that perceiving stress as negative stress is important for the adverse effects of psychosocial stress on cardiovascular health (115).

In epidemiological studies, effect sizes for the relationship between psychosocial stress and CVD have been statistically corrected for other variables. However, chronic stress may exacerbate the risk for CVD even further by promoting lifestyle factors that are themselves risk factors for CVD such as smoking, sedentary lifestyle, unhealthy diet, or disturbed sleep (75, 122).

Observational studies not only link chronic psychosocial stress to CVD in general but also suggest a contribution by inflammatory processes. In this regard, higher levels of clinical proinflammatory risk markers such as TNFα, IL-6, C-reactive protein (CRP), and soluble adhesion molecules were found in plasma samples from individuals with a high burden of chronic stress (68, 73, 83). Similarly, a study in medical residents on an intensive care unit found increased levels of blood inflammatory leukocytes when residents were on duty compared with when they were off duty (57). Using state-of-the-art imaging techniques, researchers also showed an association between resting amygdala activity—the amygdala is part of the brain's limbic system and processes emotional responses—reflecting the degree of perceived stress and the risk for cardiovascular events (164). On top of that, amygdala activity was directly connected with bone marrow activity, reflecting hematopoiesis, and arterial inflammation (69, 164). Further evidence for the influence of chronic psychosocial stress on cardiovascular inflammation in humans is available from intervention trials, which consistently show reduced inflammatory activation in response to immune challenges following stress-reducing interventions (144).

**FIG. 5.**
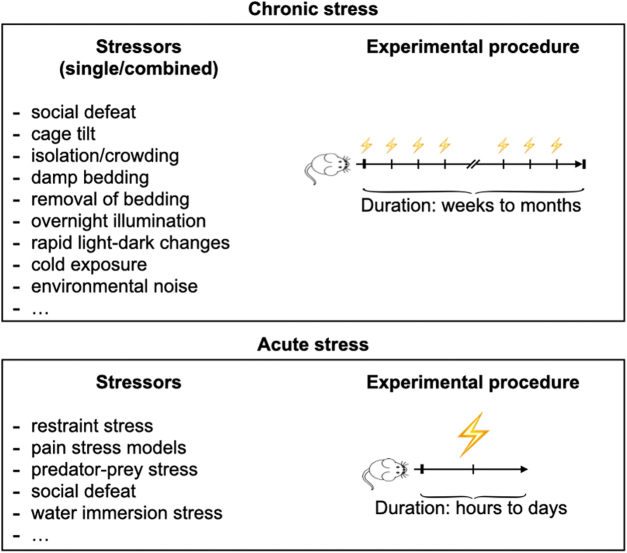
**Experimental mouse models to induce psychosocial stress.** In experimental models for chronic stress, animals are exposed to a single mild stressor or a combination of mild stressors repetitively for weeks up to months. By contrast, experimental models for acute stress use severe stressors over a short period of time (hours to days). 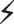, Stress exposure.

Findings in animal studies may reveal the mechanistic processes underlying the adverse effect of chronic psychosocial stress on vascular inflammation; hence, data from animal studies are inevitable. Attaining useful accurate results requires proper experimental models for both atherosclerosis and the simulation of chronic psychosocial stress. Classically, atherosclerosis experiments are done in animals with specific genetic knockouts that lead to hypercholesteremia and consequently result in the development of lesions similar to human atherosclerosis. Due to the need for genetic modification and their advantageously short generation times, mouse models are predominantly used (46). Most such mouse models harbor a knockout in either the apolipoprotein E (*ApoE*) gene or the *LDL* gene, and both varieties develop hyperlipidemia and vascular inflammation when fed a cholesterol-enriched diet. Models for simulating chronic psychosocial stress in animals are numerous and were mostly established by behavioral neuroscience studies (120, 147). In chronic stress models, animals are exposed to a single mild stressor or a combination of mild stressors repetitively over a longer time period (83). Social isolation and defeat, disrupted circadian rhythm, and environmental noise are just a few examples of the wide range of unpleasant situations that are used as stressors in animal studies (Fig. 5).

As described above, chronic psychosocial stress is associated with higher levels of inflammatory markers in humans. This has been confirmed by animal studies that demonstrated the presence of systemic low-grade inflammation as a result of chronic psychosocial stress (6, 104). Likewise, chronic psychosocial stress increased circulating inflammatory leukocyte levels in an animal model of chronic stress (57). This increase is reportedly caused by the SNS overactivity in the bone marrow, subsequently leading to increased hematopoietic stem cell proliferation and release of neutrophils and inflammatory monocytes into the blood stream. Leukocytes not only undergo quantitative changes but also alter their phenotypes qualitatively in response to chronic stress. A study using repeated social defeat demonstrated that circulating leukocytes acquire a proinflammatory gene signature in response to chronic stress, hand in hand with a bias toward myelopoiesis in bone marrow hematopoiesis (126).

Another phenomenon that applies in chronic psychosocial stress is the concept of trained immunity. It describes the observation that not only the adaptive immune system but also the innate immune system develops immunological memory to previous challenges (119). Immunological memory is conveyed by both epigenetic and metabolic reprogramming in innate immune cells (37). In first studies, trained immunity was shown to play a role in the response to immune challenges after initial priming with stress hormones. Monocytes that were exposed to high levels of norepinephrine and subsequently differentiated to macrophages *in vitro* mounted a heightened proinflammatory response after restimulation with lipopolysaccharide (LPS) (58). Accordingly, a murine macrophage-like cell line secreted higher amounts of IL-6 and TNFα when primed with glucocorticoids before LPS stimulation (156).

Inflammation in chronic stress situations affects not only leukocytes but also their counterparts in the process of leukocyte recruitment: endothelial cells. It is well known that circulating cytokines activate endothelial cells (11, 96) and, as outlined above, chronic psychosocial stress strongly contributes to systemic low-grade inflammation. Mechanistically, most cytokines upregulate adhesion molecule expression on endothelial cells, a change that subsequently leads to more leukocyte recruitment in the underlying tissue (24, 127). Furthermore, systemic activation of the renin/angiotensin/aldosterone system during chronic psychosocial stress may contribute to endothelial dysfunction and cardiovascular inflammation (148). The renin/angiotensin/aldosterone system involves a multitude of players, such as angiotensin II, that act jointly to regulate fluid balance and blood pressure. Angiotensin II, similar to systemic proinflammatory cytokines, induces increased expression of adhesion molecules, chemokines, and cytokines by endothelial cells (50, 99). Beyond that, endothelial cells express receptors for all major stress hormones (139, 193) and may thus be activated directly by elevated circulating hormone levels.

Taken together, the abovementioned experimental studies demonstrate the proinflammatory potential of psychosocial stress. Indeed, various experimental evidences demonstrate accelerated atherosclerosis development in chronic stress models (83). Reports about changes in lesion size are inconsistent (9, 10), however, researchers agree that chronic psychosocial stress changes the plaque phenotype toward a more unstable lesion, which includes more plaque inflammatory leukocytes (57, 191, 192), fewer plaque SMCs (191, 192), higher expression levels of matrix metalloproteinases (57, 192) and, consequently, reduced plaque collagen content (192) and a thinner fibrous cap (118, 191). Of note, standard murine models of atherosclerosis acquire features of unstable plaques but lack plaque rupture or erosion, which frequently occur as the ultimate consequences of atherosclerosis in humans. Using additional gene knockouts or surgical techniques, researchers were recently able to mimic these conditions in mice (152). In an *ApoE*(−/−)*Fbn1*(C1039G+/−) mouse model that develops exacerbated atherosclerosis and spontaneous plaque ruptures, chronic psychosocial stress indeed increased plaque instability and the incidence of MI (137).

Overall, evidence for the proinflammatory effect of chronic psychosocial stress has expanded greatly in recent years, with general consensus regarding its capacity to promote vascular inflammation and atherosclerosis progression. Mechanistically, this is one aspect of the links between chronic stress and higher CVD risk in affected patients.

##### Acute psychosocial stress

In contrast to chronic stress, which increases the risk for CVD gradually over time, acute stress is a major trigger for acute cardiovascular events in people with manifest atherosclerosis (106, 159). In recent decades, natural catastrophes, such as earthquakes, or other population-level disasters, such as war and terror attacks, have been associated with higher incidences of acute cardiovascular events (4, 103). Following an earthquake in the Los Angeles area in 1994, for example, the occurrence of sudden deaths from cardiac causes rose around fivefold compared with control periods before the earthquake and in the preceding years (88). However, even more moderate stressors such as major sporting events have been linked to cardiovascular complications (181).

The notion that acute stress can trigger acute cardiac events is further supported by a great number of individual-level studies (34). A recent meta-analysis calculated a 4.74 higher risk for MI or acute coronary syndrome within 2 h following anger, for example (108). This was confirmed in a subanalysis of the INTERHEART study that reported a 2.44 higher odds ratio for acute MI associated with emotional upset (155).

Observational studies show that, similar to chronic psychosocial stress, acute stress is linked to inflammation (83). In a study investigating the effect of a stressful sporting event, classical inflammatory mediators such as soluble vascular adhesion molecule 1 (sVCAM-1), monocyte chemoattractant protein 1 (MCP-1), and TNFα were up to 65% higher in subjects experiencing a stress-associated ACS event compared with control patients (182). Likewise, patients suffering from Takotsubo cardiomyopathy, an acute stress-associated type of nonischemic cardiomyopathy, displayed higher plasma levels of inflammatory cytokines (143).

Experimental human data provided further evidence that acute stress produces proinflammatory responses in the body. Psychological research experiments involving public speaking tests, arithmetic tasks, or a combination of different stressors were the first simulations of acute psychosocial stress in humans. In a study using oral presentations as a stress model in physicians, participants had increased plasma levels of IL-1ꞵ and ICAM-1 (59). A classical adhesion molecule on endothelial cells, ICAM-1 mediates leukocyte recruitment and is upregulated under inflammatory conditions. Its soluble form is also found in plasma and serves as a biomarker for atherosclerotic plaque burden (5). In another study using a standardized public speaking stressor, greater peripheral vasoconstriction with mental stress was associated with a higher risk of adverse cardiovascular outcomes in participants with preexisting CVD (74). Of note, the researchers observed an association between greater peripheral vasoconstriction and circulating norepinephrine and IL-6 plasma levels. An interesting recent study showed that increased mental stress-induced myocardial ischemia in young women post-MI is accompanied by more microvascular dysfunction than in their male counterparts (168). Importantly, microvascular dysfunction has been linked to systemic inflammation by several studies (36, 130, 131). In a randomized-controlled trial using a sequential series of psychosocial stressors, acute stress raised the plasma levels of IL-6 and IL-1ꞵ (80). In addition, this rise in inflammatory cytokines was accompanied by a proinflammatory gene expression profile in blood leukocytes, an effect likely mediated by the transcription factor nuclear factor kappa B (NF-κB). Another recent randomized-controlled trial study reported increased IL-6 levels in a CO_2_ stress test that simulated acute stress in humans (76). This test was previously established as a more severe experimental model for panic disorders (172).

While chronic psychosocial stress consistently elevated circulating leukocytes and inflammatory subtypes in human and animal studies (57), things are less clear in acute psychosocial stress. On the one hand, for example, performing a speech task resulted in general leukocytosis and higher levels of all major leukocyte subtypes (48). On the other hand, a study that used skydiving as a model for acute psychosocial stress reported increased neutrophils and natural killer cells, while lymphocyte and monocyte levels decreased immediately before and after the jump compared with baseline (14). These differences may be explained by the different kinetics of leukocyte populations and varied stress stimuli used in studies. The picture becomes even more complex as the acute stress response redistributes leukocyte subsets between hematopoietic organs, blood, and various tissues (62).

As with chronic psychosocial stress, detailed mechanistic data on how acute stress exacerbates disease come from animal and cell culture experiments. To simulate acute stress, studies use severe stressors including restraint stress (128, 175, 187), in which animals are immobilized for minutes up to several hours, pain stress models such as eye bleeding (128) or social defeat (Fig. 5) (128, 187).

Similar to human experimental data, animal studies demonstrate that an acute stress procedure impacts circulating blood leukocyte levels. However, in contrast to human data, individual studies using animal models have more aligned outcomes, mostly likely due to better standardization in animal experiments. Such studies consistently report a decline in circulating monocyte and lymphocyte levels after at least 30 min of acute stress, while neutrophil levels are unchanged or even increase (61, 175, 186, 187). Initially, leukocytes are massively mobilized from reservoirs, but blood numbers rapidly decline as leukocytes traffic to sites of inflammation and immune activation (25).

This redistribution also requires endothelial cell compartment activation in the respective tissue. However, although researchers show that psychosocial stress robustly affects the endothelium in the context of hemodynamic processes (40, 125, 168), very little is known about the inflammatory consequences. Our group recently proved that locally released norepinephrine activates endothelial cells under acute stress exposure (61). As a consequence, activated endothelial cells increasingly express cell adhesion molecules and release chemokines, which result in enhanced blood inflammatory leukocyte recruitment to susceptible tissues, especially atherosclerotic plaques. In addition, similar to chronic stress situations, the increased circulating chemokines likely further activate the endothelium in an already proinflammatory setting due to preexisting atherosclerosis. Cardiovascular events such as MI can themselves be regarded as acute psychosocial stress. Accordingly, prior MI accelerated atherosclerosis in an animal model (31).

As plaque rupture does not often occur in classical, atherosclerotic animal models, research previously focused primarily on how acute psychosocial stress impacted plaque phenotypes, cardiac function, and hemostatic processes (83). Researchers showed that acute psychosocial stress leads to plaque destabilization (84) and ultimately MI in hypercholesterolemic *ApoE*^−/−^ mice (16). Indeed, our group recently showed that acute mental stress affects plaque stability in a mouse plaque rupture model. Mechanistically, increased plaque rupture incidence was accompanied by higher intimal myeloid cell numbers and decreased SMC and collagen content (61).

As mentioned above, human data suggest an association between acute stress and systemic inflammation, and significantly accumulating recent evidence also links acute psychosocial stress to increased vascular inflammation.

## Clinical Outlook and Future Perspectives

In summary, society faces a high burden of psychosocial stress-related CVD cases. Although lipid-lowering therapy has greatly reduced CVD risk in recent decades, the residual risk remains substantial, and psychosocial factors strongly contribute to this condition (188). Indeed, both chronic stress and acute stress are associated with CVD risk, even in patients receiving state-of-the-art treatment (181).

Successful intervention in this area first requires the identification of people at risk (75). These individuals have both preexisting atherosclerosis and either a high burden of chronic stress or a behavior type that is especially susceptible to acute stress. Biomarkers such as hormone levels and platelet aggregates at baseline or after stress challenges might help identify people carrying this type of risk (160).

Behavioral interventions might be best suited to reducing chronic stress. Such interventions include both population-based and targeted approaches; the latter is mostly used in secondary prevention (75). It is worth noting that the occurrence of acute stress strongly depends on external factors and cannot necessarily be reduced by behavioral interventions. Still, possible behavioral intervention strategies may target coping mechanisms in particularly stress-susceptible individuals (52, 185) or provide external support strategies in both chronic stress and acute stress. Potential resilience and coping mechanisms that might be entrained include mindfulness, meditation, cognitive behavioral therapy, and physical activity (157, 179). Up to now, however, systematic analysis of such interventions in controlled trials is lacking (75).

**FIG. 6.**
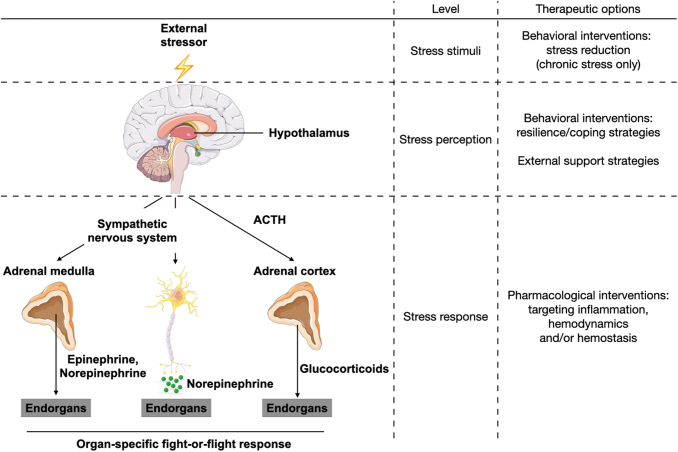
**Therapeutic options of psychosocial stress.** People at high risk of stress-induced CVD can potentially be treated at three different levels alongside the physiological stress response cascade. Especially in chronic stress, the stimulus itself can be removed/reduced. On the stress perception level, behavioral intervention strategies may increase resilience to stressful events or strengthen coping strategies. In addition, external support strategies by the individual environment can be established. Pharmacological interventions to modify the stress response itself can target inflammatory processes (as outlined in this review), and also hemodynamics and hemostasis. However, up to now, pharmacological interventions in the context of psychosocial stress and CVD are not routinely used in clinical practice. CVD, cardiovascular disease.

Along with behavioral interventions, susceptible individuals may need pharmacological treatment (Fig. 6). However, there are currently no drugs that reduce chronic or acute stress-associated inflammation, partly due to a paucity of mechanistic understanding. In chronic stress, the specific effects of stress hormones on endothelial cells and intraplaque processes particularly need to be addressed. For the acute stress response, different questions remain unanswered. It is still largely unknown whether acute mental stress influences other vascular cell types apart from endothelial cells. Furthermore, the effects of acute mental stress on circulating leukocytes warrant further investigation. Most of all, tailoring pharmacological interventions requires a deeper knowledge of stress-specific mechanistic effects on inflammation. The majority of circulating cytokines that are upregulated during psychosocial stress, for example, IL-1ꞵ and IL-6, are also crucial players in the immune response to nonsterile infections. Thus, targeting these cytokines in stress therapy may lead to off-target effects and increase, for instance, the incidence of fatal infections.

All in all, psychosocial factors are increasingly acknowledged as key to the primary and secondary prevention of CVD. Going forward, additional mechanistic studies are needed to provide better tools for behavioral and pharmacological treatment options.

## Abbreviations Used

ACTH: adrenocorticotropic hormone
ApoE: apolipoprotein E
CCL: CC-chemokine ligand
CD: cluster of differentiation
CVD: cardiovascular disease
DFG: Deutsche Forschungsgemeinschaft
HPA: hypothalamus/pituitary/adrenal
ICAM-1: intercellular adhesion molecule 1
IL: interleukin
LDL: low-density lipoprotein
LPS: lipopolysaccharide
MI: myocardial infarction
PBMC: peripheral blood mononuclear cell
RA: rheumatoid arthritis
SMC: smooth muscle cell
SNS: sympathetic nervous system
TNFα: tumor necrosis factor alpha

## References

[1] Abbate A, Toldo S, Marchetti C, Kron J, Tassell BWV, and Dinarello CA. Interleukin-1 and the inflammasome as therapeutic targets in cardiovascular disease. Circ Res 126: 1260–1280, 2020. 32324502 10.1161/CIRCRESAHA.120.315937 PMC8760628

[2] Adamstein NH, MacFadyen JG, Rose LM, Glynn RJ, Dey AK, Libby P, Tabas IA, Mehta NN, and Ridker PM. The neutrophil–lymphocyte ratio and incident atherosclerotic events: analyses from five contemporary randomized trials. Eur Heart J 42: 896–903, 2021. 33417682 10.1093/eurheartj/ehaa1034 PMC7936519

[3] Ait-Oufella H, Herbin O, Bouaziz J-D, Binder CJ, Uyttenhove C, Laurans L, Taleb S, Vré EV, Esposito B, Vilar J, Sirvent J, Snick JV, Tedgui A, Tedder TF, and Mallat Z. B cell depletion reduces the development of atherosclerosis in mice. J Exp Med 207: 1579–1587, 2010. 20603314 10.1084/jem.20100155 PMC2916123

[4] Aoki T, Fukumoto Y, Yasuda S, Sakata Y, Ito K, Takahashi J, Miyata S, Tsuji I, and Shimokawa H. The Great East Japan Earthquake Disaster and cardiovascular diseases. Eur Heart J 33: 2796–2803, 2012. 22930461 10.1093/eurheartj/ehs288

[5] Ballantyne CM and Entman ML. Soluble adhesion molecules and the search for biomarkers for atherosclerosis. Circulation 106: 766–767, 2002. 12176941 10.1161/01.cir.0000028397.68936.12

[6] Barnum CJ, Pace TWW, Hu F, Neigh GN, and Tansey MG. Psychological stress in adolescent and adult mice increases neuroinflammation and attenuates the response to LPS challenge. J Neuroinflammation 9: 15, 2012. 22248083 10.1186/1742-2094-9-9 PMC3283491

[7] Barrett TJ. Macrophages in atherosclerosis regression. Arterioscler Thromb Vasc Biol 40: 20–33, 2018. 10.1161/ATVBAHA.119.312802 PMC6946104 31722535

[8] Berg KE, Ljungcrantz I, Andersson L, Bryngelsson C, Hedblad B, Fredrikson GN, Nilsson J, and Björkbacka H. Elevated CD14^++^CD16^−^ monocytes predict cardiovascular events. Circ Cardiovasc Genet 5: 122–131, 2012. 22238190 10.1161/CIRCGENETICS.111.960385

[9] Bernberg E, Andersson IJ, Tidstrand S, Johansson ME, and Bergström G. Repeated exposure to stressors do not accelerate atherosclerosis in ApoE^−/−^ mice. Atherosclerosis 204: 90–95, 2009. 18834587 10.1016/j.atherosclerosis.2008.08.013

[10] Bernberg E, Ulleryd MA, Johansson ME, and Bergström GML. Social disruption stress increases IL-6 levels and accelerates atherosclerosis in ApoE^−/−^ mice. Atherosclerosis 221: 359–365, 2012. 22284955 10.1016/j.atherosclerosis.2011.11.041

[11] Bevilacqua MP, Pober JS, Wheeler ME, Cotran RS, and Gimbrone MA. Interleukin-1 activation of vascular endothelium. Effects on procoagulant activity and leukocyte adhesion. Am J Pathol 121: 394–403, 1985. 3878084 PMC1887931

[12] Bienertova-Vasku J, Lenart P, and Scheringer M. Eustress and distress: neither good nor bad, but rather the same? Bioessays 42: 1900238, 2020. 10.1002/bies.201900238 32302008

[13] Bras AL. Poor sleep linked to atherosclerosis. Nat Rev Cardiol 16: 132–132, 2019. 10.1038/s41569-019-0162-9 30696979

[14] Breen MS, Beliakova-Bethell N, Mujica-Parodi LR, Carlson JM, Ensign WY, Woelk CH, and Rana BK. Acute psychological stress induces short-term variable immune response. Brain Behav Immun 53: 172–182, 2016. 26476140 10.1016/j.bbi.2015.10.008

[15] Cai L, Wheeler E, Kerrison ND, Luan J, Deloukas P, Franks PW, Amiano P, Ardanaz E, Bonet C, Fagherazzi G, Groop LC, Kaaks R, Huerta JM, Masala G, Nilsson PM, Overvad K, Pala V, Panico S, Rodriguez-Barranco M, Rolandsson O, Sacerdote C, Schulze MB, Spijkerman AMW, Tjonneland A, Tumino R, van der Schouw YT, Sharp SJ, Forouhi NG, Riboli E, McCarthy MI, Barroso I, Langenberg C, and Wareham NJ. Genome-wide association analysis of type 2 diabetes in the EPIC-InterAct study. Sci Data 7: 393, 2020. 33188205 10.1038/s41597-020-00716-7 PMC7666191

[16] Caligiuri G, Levy B, Pernow J, Thorén P, and Hansson GK. Myocardial infarction mediated by endothelin receptor signaling in hypercholesterolemic mice. Proc Natl Acad Sci U S A 96: 6920–6924, 1999. 10359814 10.1073/pnas.96.12.6920 PMC22017

[17] Caligiuri G, Nicoletti A, Poirier B, and Hansson GK. Protective immunity against atherosclerosis carried by B cells of hypercholesterolemic mice. J Clin Invest 109: 745–753, 2002. 11901183 10.1172/JCI07272 PMC150903

[17a] CARDIoGRAMplusC4D Consortium; Deloukas P, Kanoni S, Willenborg C, Farrall M, Assimes TL, Thompson JR, Ingelsson E, Saleheen D, Erdmann J, Goldstein BA, Stirrups K, König IR, Cazier J-B, Johansson Å, Hall AS, Lee J-Y, Willer CJ, Chambers JC, Esko T, Folkersen L, Goel A, Grundberg E, Havulinna AS, Ho WK, Hopewell JC, Eriksson N, Kleber ME, Kristiansson K, Lundmark P, Lyytikäinen L-P, Rafelt S, Shungin D, Strawbridge RJ, Thorleifsson G, Tikkanen E, Zuydam NV, Voight BF, Waite LL, Zhang W, Ziegler A, Absher D, Altshuler D, Balmforth AJ, Barroso I, Braund PS, Burgdorf C, Claudi-Boehm S, Cox D, Dimitriou M, Do R, DI AGRAM Consortium; CARDIOGENICS Consortium; Doney ASF, Mokhtari NE, Eriksson P, Fischer K, Fontanillas P, Franco-Cereceda A, Gigante B, Groop L, Gustafsson S, Hager J, Hallmans G, Han B-G, Hunt SE, Kang HM, Illig T, Kessler T, Knowles JW, Kolovou G, Kuusisto J, Langenberg C, Langford C, Leander K, Lokki M-L, Lundmark A, McCarthy MI, Meisinger C, Melander O, Mihailov E, Maouche S, Morris AD, Müller-Nurasyid M, MuTHER Consortium; Nikus K, Peden JF, Rayner NW, Rasheed A, Rosinger S, Rubin D, Rumpf MP, Schäfer A, Sivananthan M, Song C, Stewart AFR, Tan S-T, Thorgeirsson G, Schoot CE van der, Wagner PJ, Wells GA, Wild PS, Yang T-P, Amouyel P, Arveiler D, Basart H, Boehnke M, Boerwinkle E, Brambilla P, Cambien F, Cupples AL, Faire U de, Dehghan A, Diemert P, Epstein SE, Evans A, Ferrario MM, Ferrières J, Gauguier D, Go AS, Goodall AH, Gudnason V, Hazen SL, Holm H, Iribarren C, Jang Y, Kähönen M, Kee F, Kim H-S, Klopp N, Koenig W, Kratzer W, Kuulasmaa K, Laakso M, Laaksonen R, Lee J-Y, Lind L, Ouwehand WH, Parish S, Park JE, Pedersen NL, Peters A, Quertermous T, Rader DJ, Salomaa V, Schadt E, Shah SH, Sinisalo J, Stark K, Stefansson K, Trégouët D-A, Virtamo J, Wallentin L, Wareham N, Zimmermann ME, Nieminen MS, Hengstenberg C, Sandhu MS, Pastinen T, Syvänen A-C, Hovingh GK, Dedoussis G, Franks PW, Lehtimäki T, Metspalu A, Zalloua PA, Siegbahn A, Schreiber S, Ripatti S, Blankenberg SS, Perola M, Clarke R, Boehm BO, O'Donnell C, Reilly MP, März W, Collins R, Kathiresan S, Hamsten A, Kooner JS, Thorsteinsdottir U, Danesh J, Palmer CNA, Roberts R, Watkins H, Schunkert H, and Samani NJ. Large-scale association analysis identifies new risk loci for coronary artery disease. Nat Genet 45: 25–33, 2013. 23202125 10.1038/ng.2480 PMC3679547

[18] Carnevale D and Lembo G. Neuroimmune interactions in cardiovascular diseases. Cardiovasc Res 117: 402–410, 2020. 10.1093/cvr/cvaa151 32462184

[19] Carson JAS, Lichtenstein AH, Anderson CAM, Appel LJ, Kris-Etherton PM, Meyer KA, Petersen K, Polonsky T, Horn LV, American Heart Association Nutrition Committee of the Council on Lifestyle and Cardiometabolic Health; Council on Arteriosclerosis, Thrombosis and Vascular Biology; Council on Cardiovascular and Stroke Nursing; Council on Clinical Cardiology; Council on Peripheral Vascular Disease; and Stroke Council. Dietary cholesterol and cardiovascular risk: a science advisory from the American Heart Association. Circulation 141: e39–e53, 2019. 31838890 10.1161/CIR.0000000000000743

[20] Chen J, Rodopoulou S, Hoogh K de, Strak M, Andersen ZJ, Atkinson R, Bauwelinck M, Bellander T, Brandt J, Cesaroni G, Concin H, Fecht D, Forastiere F, Gulliver J, Hertel O, Hoffmann B, Hvidtfeldt UA, Janssen NAH, Jöckel K-H, Jørgensen J, Katsouyanni K, Ketzel M, Klompmaker JO, Lager A, Leander K, Liu S, Ljungman P, MacDonald CJ, Magnusson PKE, Mehta A, Nagel G, Oftedal B, Pershagen G, Peters A, Raaschou-Nielsen O, Renzi M, Rizzuto D, Samoli E, van der Schouw YT, Schramm S, Schwarze P, Sigsgaard T, Sørensen M, Stafoggia M, Tjønneland A, Vienneau D, Weinmayr G, Wolf K, Brunekreef B, and Hoek G. Long-term exposure to fine particle elemental components and natural and cause-specific mortality—a pooled analysis of eight European cohorts within the ELAPSE project. Environ Health Perspect 129: 47009, 2021. 33844598 10.1289/EHP8368 PMC8041432

[21] Combadière C, Potteaux S, Rodero M, Simon T, Pezard A, Esposito B, Merval R, Proudfoot A, Tedgui A, and Mallat Z. Combined inhibition of CCL2, CX3CR1, and CCR5 abrogates Ly6Chi and Ly6Clo monocytosis and almost abolishes atherosclerosis in hypercholesterolemic mice. Circulation 117: 1649–1657, 2008. 18347211 10.1161/CIRCULATIONAHA.107.745091

[22] This reference has been deleted.

[23] Daiber A, Lelieveld J, Steven S, Oelze M, Kröller-Schön S, Sørensen M, and Münzel T. The “exposome” concept—how environmental risk factors influence cardiovascular health. Acta Biochim Pol 66: 269–283, 2019. 31509369 10.18388/abp.2019_2853

[24] Deng X, Chu X, Wang P, Ma X, Wei C, Sun C, Yang J, and Li Y. MicroRNA-29a-3p reduces TNFα-induced endothelial dysfunction by targeting tumor necrosis factor receptor 1. Mol Ther Nucleic Acids 18: 903–915, 2019. 31760375 10.1016/j.omtn.2019.10.014 PMC6883339

[25] Dhabhar FS, Malarkey WB, Neri E, and McEwen BS. Stress-induced redistribution of immune cells—from barracks to boulevards to battlefields: a tale of three hormones—Curt Richter Award Winner. Psychoneuroendocrinology 37: 1345–1368, 2012. 22727761 10.1016/j.psyneuen.2012.05.008 PMC3412918

[26] Dimsdale JE. Psychological stress and cardiovascular disease. J Am Coll Cardiol 51: 1237–1246, 2008. 18371552 10.1016/j.jacc.2007.12.024 PMC2633295

[27] Döring Y, Drechsler M, Soehnlein O, and Weber C. Neutrophils in atherosclerosis. Arterioscler Thromb Vasc Biol 35: 288–295, 2015. 25147339 10.1161/ATVBAHA.114.303564

[28] Drechsler M, Duchene J, and Soehnlein O. Chemokines control mobilization, recruitment, and fate of monocytes in atherosclerosis. Arterioscler Thromb Vasc Biol 35: 1050–1055, 2018. 10.1161/ATVBAHA.114.304649 25792446

[29] Drechsler M, Megens RTA, van Zandvoort M, Weber C, and Soehnlein O. Hyperlipidemia-triggered neutrophilia promotes early atherosclerosis. Circulation 122: 1837–1845, 2010. 20956207 10.1161/CIRCULATIONAHA.110.961714

[30] Duewell P, Kono H, Rayner KJ, Sirois CM, Vladimer G, Bauernfeind FG, Abela GS, Franchi L, Nuñez G, Schnurr M, Espevik T, Lien E, Fitzgerald KA, Rock KL, Moore KJ, Wright SD, Hornung V, and Latz E. NLRP3 inflammasomes are required for atherogenesis and activated by cholesterol crystals. Nature 464: 1357–1361, 2010. 20428172 10.1038/nature08938 PMC2946640

[31] Dutta P, Courties G, Wei Y, Leuschner F, Gorbatov R, Robbins CS, Iwamoto Y, Thompson B, Carlson AL, Heidt T, Majmudar MD, Lasitschka F, Etzrodt M, Waterman P, Waring MT, Chicoine AT, Laan AM van der, Niessen HWM, Piek JJ, Rubin BB, Butany J, Stone JR, Katus HA, Murphy SA, Morrow DA, Sabatine MS, Vinegoni C, Moskowitz MA, Pittet MJ, Libby P, Lin CP, Swirski FK, Weissleder R, and Nahrendorf M. Myocardial infarction accelerates atherosclerosis. Nature 487: 325–329, 2012. 22763456 10.1038/nature11260 PMC3401326

[32] Dutta P, Sager HB, Stengel KR, Naxerova K, Courties G, Saez B, Silberstein L, Heidt T, Sebas M, Sun Y, Wojtkiewicz G, Feruglio PF, King K, Baker JN, van der Laan AM, Borodovsky A, Fitzgerald K, Hulsmans M, Hoyer F, Iwamoto Y, Vinegoni C, Brown D, Di Carli M, Libby P, Hiebert SW, Scadden DT, Swirski FK, Weissleder R, and Nahrendorf M. Myocardial infarction activates CCR2^+^ hematopoietic stem and progenitor cells. Cell Stem Cell 16: 477–487, 2015. 25957903 10.1016/j.stem.2015.04.008 PMC4426344

[33] Ebner K and Singewald N. Individual differences in stress susceptibility and stress inhibitory mechanisms. Curr Opin Behav Sci 14: 54–64, 2017.

[34] Edmondson D, Newman JD, Whang W, and Davidson KW. Emotional triggers in myocardial infarction: do they matter? Eur Heart J 34: 300–306, 2012. 23178642 10.1093/eurheartj/ehs398 PMC3549526

[35] Estruch R, Ros E, Salas-Salvadó J, Covas M-I, Corella D, Arós F, Gómez-Gracia E, Ruiz-Gutiérrez V, Fiol M, Lapetra J, Lamuela-Raventos RM, Serra-Majem L, Pintó X, Basora J, Muñoz MA, Sorlí JV, Martínez JA, and Martínez-González MA; PREDIMED Study Investigators. Primary prevention of cardiovascular disease with a Mediterranean diet. N Engl J Med 368: 1279–1290, 2013. 29897867 10.1056/NEJMc1806491

[36] Faccini A, Kaski JC, and Camici PG. Coronary microvascular dysfunction in chronic inflammatory rheumatoid diseases. Eur Heart J 37: 1799–1806, 2016. 26912605 10.1093/eurheartj/ehw018

[37] Fanucchi S, Domínguez-Andrés J, Joosten LAB, Netea MG, and Mhlanga MM. The intersection of epigenetics and metabolism in trained immunity. Immunity 54: 32–43, 2020. 33220235 10.1016/j.immuni.2020.10.011

[38] Fernandez DM, Rahman AH, Fernandez NF, Chudnovskiy A, Amir ED, Amadori L, Khan NS, Wong CK, Shamailova R, Hill CA, Wang Z, Remark R, Li JR, Pina C, Faries C, Awad AJ, Moss N, Bjorkegren JLM, Kim-Schulze S, Gnjatic S, Ma'ayan A, Mocco J, Faries P, Merad M, and Giannarelli C. Single-cell immune landscape of human atherosclerotic plaques. Nat Med 25: 1576–1588, 2019. 31591603 10.1038/s41591-019-0590-4 PMC7318784

[39] Floud S, Blangiardo M, Clark C, Hoogh K de, Babisch W, Houthuijs D, Swart W, Pershagen G, Katsouyanni K, Velonakis M, Vigna-Taglianti F, Cadum E, and Hansell AL. Exposure to aircraft and road traffic noise and associations with heart disease and stroke in six European countries: a cross-sectional study. Environ Health 12: 89, 2013. 24131577 10.1186/1476-069X-12-89 PMC4015897

[40] Fox BM, Becker BK, Loria AS, Hyndman KA, Jin C, Clark H, Johns R, Yanagisawa M, Pollock DM, and Pollock JS. Acute pressor response to psychosocial stress is dependent on endothelium-derived endothelin-1. J Am Heart Assoc 7: e007863, 2018. 29453306 10.1161/JAHA.117.007863 PMC5850198

[41] Franck G, Mawson TL, Folco EJ, Molinaro R, Ruvkun V, Engelbertsen D, Liu X, Tesmenitsky Y, Shvartz E, Sukhova GK, Michel J-B, Nicoletti A, Lichtman A, Wagner D, Croce KJ, and Libby P. Roles of PAD4 and NETosis in experimental atherosclerosis and arterial injury. Circ Res 123: 33–42, 2018. 29572206 10.1161/CIRCRESAHA.117.312494 PMC6014872

[42] Frodermann V, Rohde D, Courties G, Severe N, Schloss MJ, Amatullah H, McAlpine CS, Cremer S, Hoyer FF, Ji F, Koeverden ID van, Herisson F, Honold L, Masson GS, Zhang S, Grune J, Iwamoto Y, Schmidt SP, Wojtkiewicz GR, Lee I-H, Gustafsson K, Pasterkamp G, Jager SCA de, Sadreyev RI, MacFadyen J, Libby P, Ridker P, Scadden DT, Naxerova K, Jeffrey KL, Swirski FK, and Nahrendorf M. Exercise reduces inflammatory cell production and cardiovascular inflammation via instruction of hematopoietic progenitor cells. Nat Med 25: 1761–1771, 2019. 31700184 10.1038/s41591-019-0633-x PMC6858591

[43] Fuster JJ, MacLauchlan S, Zuriaga MA, Polackal MN, Ostriker AC, Chakraborty R, Wu C-L, Sano S, Muralidharan S, Rius C, Vuong J, Jacob S, Muralidhar V, Robertson AAB, Cooper MA, Andrés V, Hirschi KK, Martin KA, and Walsh K. Clonal hematopoiesis associated with TET2 deficiency accelerates atherosclerosis development in mice. Science 355: 842–847, 2017. 28104796 10.1126/science.aag1381 PMC5542057

[44] Galkina E, Kadl A, Sanders J, Varughese D, Sarembock IJ, and Ley K. Lymphocyte recruitment into the aortic wall before and during development of atherosclerosis is partially L-selectin dependent. J Exp Med 203: 1273–1282, 2006. 16682495 10.1084/jem.20052205 PMC2121208

[45] Gaul DS, Stein S, and Matter CM. Neutrophils in cardiovascular disease. Eur Heart J 38: 1702–1704, 2017. 30052884 10.1093/eurheartj/ehx244

[46] Getz GS and Reardon CA. Animal models of atherosclerosis. Arterioscler Thromb Vasc Biol 32: 1104–1115, 2018. 10.1161/ATVBAHA.111.237693 PMC3331926 22383700

[46a] Giri A, Hellwege JN, Keaton JM, Park J, Qiu C, Warren HR, Torstenson ES, Kovesdy CP, Sun YV, Wilson OD, Robinson-Cohen C, Roumie CL, Chung CP, Birdwell KA, Damrauer SM, DuVall SL, Klarin D, Cho K, Wang Y, Evangelou E, Cabrera CP, Wain LV, Shrestha R, Mautz BS, Akwo EA, Sargurupremraj M, Debette S, Boehnke M, Scott LJ, Luan J, Zhao J-H, Willems SM, Thériault S, Shah N, Oldmeadow C, Almgren P, Li-Gao R, Verweij N, Boutin TS, Mangino M, Ntalla I, Feofanova E, Surendran P, Cook JP, Karthikeyan S, Lahrouchi N, Liu C, Sepúlveda N, Richardson TG, Kraja A, Amouyel P, Farrall M, Poulter NR, Understanding Society Scientific Group; International Consortium for Blood Presssure; Blood Pressure-International Consortium of Exome Chip Studies; Laakso M, Zeggini E, Sever P, Scott RA, Langenberg C, Wareham NJ, Conen D, Palmer CNA, Attia J, Chasman DI, Ridker PM, Melander O, Mook-Kanamori DO, Harst P van der, Cucca F, Schlessinger D, Hayward C, Spector TD, Jarvelin M-R, Hennig BJ, Timpson NJ, Wei W-Q, Smith JC, Xu Y, Matheny ME, Siew EE, Lindgren C, Herzig K-H, Dedoussis G, Denny JC, Psaty BM, Howson JMM, Munroe PB, Newton-Cheh C, Caulfield MJ, Elliott P, Gaziano JM, Concato J, Wilson PWF, Tsao PS, Edwards DRV, Susztak K, Million Veteran Program; O'Donnell CJ, Hung AM, and Edwards TL. Trans-ethnic association study of blood pressure determinants in over 750,000 individuals. Nat Genet 51: 51–62, 2019. 30578418 10.1038/s41588-018-0303-9 PMC6365102

[47] Glaser R and Kiecolt-Glaser JK. Stress-induced immune dysfunction: implications for health. Nat Rev Immunol 5: 243–251, 2005. 15738954 10.1038/nri1571

[48] Goebel MU, Mills PJ, Irwin MR, and Ziegler MG. Interleukin-6 and tumor necrosis factor-α production after acute psychological stress, exercise, and infused isoproterenol: differential effects and pathways. Psychosom Med 62: 591–598, 2000. 10949106 10.1097/00006842-200007000-00019

[49] Grau AJ, Boddy AW, Dukovic DA, Buggle F, Lichy C, Brandt T, and Hacke W; CAPRIE Investigators. Leukocyte count as an independent predictor of recurrent ischemic events. Stroke 35: 1147–1152, 2004. 15017013 10.1161/01.STR.0000124122.71702.64

[50] Groeschel M and Braam B. Connecting chronic and recurrent stress to vascular dysfunction: no relaxed role for the renin-angiotensin system. Am J Physiol Renal 300: F1–F10, 2011. 10.1152/ajprenal.00208.2010 20980410

[51] This reference has been deleted.

[52] Grueschow M, Stenz N, Thörn H, Ehlert U, Breckwoldt J, Maeder MB, Exadaktylos AK, Bingisser R, Ruff CC, and Kleim B. Real-world stress resilience is associated with the responsivity of the locus coeruleus. Nat Commun 12: 2275, 2021. 33859187 10.1038/s41467-021-22509-1 PMC8050280

[53] Hansson GK and Hermansson A. The immune system in atherosclerosis. Nat Immunol 12: 204–212, 2011. 21321594 10.1038/ni.2001

[54] Härdtner C, Kornemann J, Krebs K, Ehlert CA, Jander A, Zou J, Starz C, Rauterberg S, Sharipova D, Dufner B, Hoppe N, Dederichs T-S, Willecke F, Stachon P, Heidt T, Wolf D, Mühlen C von zur, Madl J, Kohl P, Kaeser R, Boettler T, Pieterman EJ, Princen HMG, Ho-Tin-Noé B, Swirski FK, Robbins CS, Bode C, Zirlik A, and Hilgendorf I. Inhibition of macrophage proliferation dominates plaque regression in response to cholesterol lowering. Basic Res Cardiol 115: 78, 2020. 33296022 10.1007/s00395-020-00838-4 PMC7725697

[55] van der Harst P and Verweij N. Identification of 64 novel genetic loci provides an expanded view on the genetic architecture of coronary artery disease. Circ Res 122: 433–443, 2018. 29212778 10.1161/CIRCRESAHA.117.312086 PMC5805277

[56] Hedrick CC. Lymphocytes in atherosclerosis. Arterioscler Thromb Vasc Biol 35: 253–257, 2018. 10.1161/ATVBAHA.114.305144 PMC4327776 25609772

[57] Heidt T, Sager HB, Courties G, Dutta P, Iwamoto Y, Zaltsman A, von zur Muhlen C, Bode C, Fricchione GL, Denninger J, Lin CP, Vinegoni C, Libby P, Swirski FK, Weissleder R, and Nahrendorf M. Chronic variable stress activates hematopoietic stem cells. Nat Med 20: 754–758, 2014. 24952646 10.1038/nm.3589 PMC4087061

[58] van der Heijden CDCC, Groh L, Keating ST, Kaffa C, Noz MP, Kersten S, van Herwaarden AE, Hoischen A, Joosten LAB, Timmers HJLM, Netea MG, and Riksen NP. Catecholamines induce trained immunity in monocytes in vitro and in vivo. Circ Res 127: 269–283, 2020. 32241223 10.1161/CIRCRESAHA.119.315800

[59] Heinz A, Hermann D, Smolka MN, Rieks M, Gräf K-J, Pöhlau D, Kuhn W, and Bauer M. Effects of acute psychological stress on adhesion molecules, interleukins and sex hormones: implications for coronary heart disease. Psychopharmacology 165: 111–117, 2003. 12417965 10.1007/s00213-002-1244-6

[60] Hilgendorf I, Swirski FK, and Robbins CS. Monocyte fate in atherosclerosis. Arterioscler Thromb Vasc Biol 35: 272–279, 2015. 25538208 10.1161/ATVBAHA.114.303565

[61] Hinterdobler J, Schott S, Jin H, Meesmann A, Steinsiek A-L, Zimmermann A-S, Wobst J, Müller P, Mauersberger C, Vilne B, Baecklund A, Chen C-S, Moggio A, Braster Q, Molitor M, Krane M, Kempf WE, Ladwig K-H, Hristov M, Hulsmans M, Hilgendorf I, Weber C, Wenzel P, Scheiermann C, Maegdefessel L, Soehnlein O, Libby P, Nahrendorf M, Schunkert H, Kessler T, and Sager HB. Acute mental stress drives vascular inflammation and promotes plaque destabilization in mouse atherosclerosis. Eur Heart J 2021. [Epub ahead of print]; DOI: 10.1093/eurheartj/ehab371. PMC8516477 34279021

[62] Ince LM, Weber J, and Scheiermann C. Control of leukocyte trafficking by stress-associated hormones. Front Immunol 9: 3143, 2019. 30687335 10.3389/fimmu.2018.03143 PMC6336915

[63] Ionita MG, van den Borne P, Catanzariti LM, Moll FL, de Vries J-PPM, Pasterkamp G, Vink A, and de Kleijn DPV. High neutrophil numbers in human carotid atherosclerotic plaques are associated with characteristics of rupture-prone lesions. Arterioscler Thromb Vasc Biol 30: 1842–1848, 2010. 20595650 10.1161/ATVBAHA.110.209296

[64] Jaiswal S and Ebert BL. Clonal hematopoiesis in human aging and disease. Science 366: eaan4673, 2019. 31672865 10.1126/science.aan4673 PMC8050831

[65] Jaiswal S, Natarajan P, Silver AJ, Gibson CJ, Bick AG, Shvartz E, McConkey M, Gupta N, Gabriel S, Ardissino D, Baber U, Mehran R, Fuster V, Danesh J, Frossard P, Saleheen D, Melander O, Sukhova GK, Neuberg D, Libby P, Kathiresan S, and Ebert BL. Clonal hematopoiesis and risk of atherosclerotic cardiovascular disease. N Engl J Med 377: 111–121, 2017. 28636844 10.1056/NEJMoa1701719 PMC6717509

[66] de Juan A, Ince LM, Pick R, Chen C-S, Molica F, Zuchtriegel G, Wang C, Zhang D, Druzd D, Hessenauer MET, Pelli G, Kolbe I, Oster H, Prophete C, Hergenhan SM, Albrecht U, Ripperger J, Montanez E, Reichel CA, Soehnlein O, Kwak BR, Frenette PS, and Scheiermann C. Artery-associated sympathetic innervation drives rhythmic vascular inflammation of arteries and veins. Circulation 140: 1100–1114, 2019. 31401849 10.1161/CIRCULATIONAHA.119.040232 PMC6756975

[67] von Känel R. Acute mental stress and hemostasis: when physiology becomes vascular harm. Thromb Res 135: S52–S55, 2015. 10.1016/S0049-3848(15)50444-1 PMC4386736 25861135

[68] von Känel R, Bellingrath S, and Kudielka BM. Association between burnout and circulating levels of pro- and anti-inflammatory cytokines in schoolteachers. J Psychosom Res 65: 51–59, 2008. 18582612 10.1016/j.jpsychores.2008.02.007

[69] Kang DO, Eo JS, Park EJ, Nam HS, Song JW, Park YH, Park SY, Na JO, Choi CU, Kim EJ, Rha S-W, Park CG, Seo HS, Kim CK, Yoo H, and Kim JW. Stress-associated neurobiological activity is linked with acute plaque instability via enhanced macrophage activity: a prospective serial 18F-FDG-PET/CT imaging assessment. Eur Heart J 42: 1883–1895, 2021. 33462618 10.1093/eurheartj/ehaa1095

[70] Katakami N. Mechanism of development of atherosclerosis and cardiovascular disease in diabetes mellitus. J Atheroscler Thromb 25: RV17014, 2017. 10.5551/jat.RV17014 PMC5770221 28966336

[71] Katayama Y, Battista M, Kao W-M, Hidalgo A, Peired AJ, Thomas SA, and Frenette PS. Signals from the sympathetic nervous system regulate hematopoietic stem cell egress from bone marrow. Cell 124: 407–421, 2006. 16439213 10.1016/j.cell.2005.10.041

[72] Ketelhuth DFJ and Hansson GK. Adaptive response of T and B cells in atherosclerosis. Circ Res 118: 668–678, 2016. 26892965 10.1161/CIRCRESAHA.115.306427

[73] Kiecolt-Glaser JK, Preacher KJ, MacCallum RC, Atkinson C, Malarkey WB, and Glaser R. Chronic stress and age-related increases in the proinflammatory cytokine IL-6. Proc Natl Acad Sci U S A 100: 9090–9095, 2003. 12840146 10.1073/pnas.1531903100 PMC166443

[74] Kim JH, Almuwaqqat Z, Hammadah M, Liu C, Ko Y-A, Lima B, Sullivan S, Alkhoder A, Abdulbaki R, Ward L, Bremner JD, Sheps DS, Raggi P, Sun YV, Shah AJ, Vaccarino V, and Quyyumi AA. Peripheral vasoconstriction during mental stress and adverse cardiovascular outcomes in patients with coronary artery disease. Circ Res 125: 874–883, 2019. 31550998 10.1161/CIRCRESAHA.119.315005 PMC7134565

[75] Kivimäki M and Steptoe A. Effects of stress on the development and progression of cardiovascular disease. Nat Rev Cardiol 15: 215–229, 2018. 29213140 10.1038/nrcardio.2017.189

[76] Koelsch S, Boehlig A, Hohenadel M, Nitsche I, Bauer K, and Sack U. The impact of acute stress on hormones and cytokines and how their recovery is affected by music-evoked positive mood. Sci Rep 6: 23008, 2016. 27020850 10.1038/srep23008 PMC4810374

[77] Koenig W. Persistent inflammatory residual risk despite aggressive cholesterol-lowering therapy: further evidence fuelling the dual target concept. Eur Heart J 41: 2962–2964, 2020. 32268369 10.1093/eurheartj/ehaa186

[78] Koudouovoh-Tripp P, Hüfner K, Egeter J, Kandler C, Giesinger JM, Sopper S, Humpel C, and Sperner-Unterweger B. Stress enhances proinflammatory platelet activity: the impact of acute and chronic mental stress. J Neuroimmune Pharmacol 16: 500–512, 2021. 32757120 10.1007/s11481-020-09945-4 PMC8087592

[79] Kröller-Schön S, Daiber A, Steven S, Oelze M, Frenis K, Kalinovic S, Heimann A, Schmidt FP, Pinto A, Kvandova M, Vujacic-Mirski K, Filippou K, Dudek M, Bosmann M, Klein M, Bopp T, Hahad O, Wild PS, Frauenknecht K, Methner A, Schmidt ER, Rapp S, Mollnau H, and Münzel T. Crucial role for Nox2 and sleep deprivation in aircraft noise-induced vascular and cerebral oxidative stress, inflammation, and gene regulation. Eur Heart J 39: 3528–3539, 2018. 29905797 10.1093/eurheartj/ehy333 PMC6174027

[80] Kuebler U, Zuccarella-Hackl C, Arpagaus A, Wolf JM, Farahmand F, von Känel R, Ehlert U, and Wirtz PH. Stress-induced modulation of NF-κB activation, inflammation-associated gene expression, and cytokine levels in blood of healthy men. Brain Behav Immun 46: 87–95, 2015. 25557189 10.1016/j.bbi.2014.12.024

[81] Kwong JC, Schwartz KL, Campitelli MA, Chung H, Crowcroft NS, Karnauchow T, Katz K, Ko DT, McGeer AJ, McNally D, Richardson DC, Rosella LC, Simor A, Smieja M, Zahariadis G, and Gubbay JB. Acute myocardial infarction after laboratory-confirmed influenza infection. N Engl J Med 378: 345–353, 2018. 29365305 10.1056/NEJMoa1702090

[82] Kyaw T, Tay C, Khan A, Dumouchel V, Cao A, To K, Kehry M, Dunn R, Agrotis A, Tipping P, Bobik A, and Toh B-H. Conventional B2 B cell depletion ameliorates whereas its adoptive transfer aggravates atherosclerosis. J Immunol 185: 4410–4419, 2010. 20817865 10.4049/jimmunol.1000033

[83] Lagraauw HM, Kuiper J, and Bot I. Acute and chronic psychological stress as risk factors for cardiovascular disease: insights gained from epidemiological, clinical and experimental studies. Brain Behav Immun 50: 18–30, 2015. 26256574 10.1016/j.bbi.2015.08.007

[84] Lagraauw HM, Wezel A, Velden D van der, Kuiper J, and Bot I. Stress-induced mast cell activation contributes to atherosclerotic plaque destabilization. Sci Rep 9: 2134, 2019. 30765859 10.1038/s41598-019-38679-4 PMC6375972

[85] Laszlo HE, McRobie ES, Stansfeld SA, and Hansell AL. Annoyance and other reaction measures to changes in noise exposure—a review. Sci Total Environ 435: 551–562, 2012. 22902956 10.1016/j.scitotenv.2012.06.112

[86] Lavie CJ, Ozemek C, Carbone S, Katzmarzyk PT, and Blair SN. Sedentary behavior, exercise, and cardiovascular health. Circ Res 124: 799–815, 2019. 30817262 10.1161/CIRCRESAHA.118.312669

[87] Lelieveld J, Klingmüller K, Pozzer A, Pöschl U, Fnais M, Daiber A, and Münzel T. Cardiovascular disease burden from ambient air pollution in Europe reassessed using novel hazard ratio functions. Eur Heart J 40: 1590–1596, 2019. 30860255 10.1093/eurheartj/ehz135 PMC6528157

[88] Leor J, Poole WK, and Kloner RA. Sudden cardiac death triggered by an earthquake. N Engl J Med 334: 413–419, 1996. 8552142 10.1056/NEJM199602153340701

[89] Ley K, Miller YI, and Hedrick CC. Monocyte and macrophage dynamics during atherogenesis. Arterioscler Thromb Vasc Biol 31: 1506–1516, 2011. 21677293 10.1161/ATVBAHA.110.221127 PMC3133596

[90] Libby P. Inflammation in atherosclerosis. Nature 420: 868–874, 2002. 12490960 10.1038/nature01323

[91] Libby P. Mechanisms of acute coronary syndromes and their implications for therapy. N Engl J Med 368: 2004–2013, 2013. 23697515 10.1056/NEJMra1216063

[92] Libby P, Buring JE, Badimon L, Hansson GK, Deanfield J, Bittencourt MS, Tokgözoğlu L, and Lewis EF. Atherosclerosis. Nat Rev Dis Primers 5: 56, 2019. 31420554 10.1038/s41572-019-0106-z

[93] Libby P, Ridker PM, and Hansson GK. Progress and challenges in translating the biology of atherosclerosis. Nature 473: 317–325, 2011. 21593864 10.1038/nature10146

[94] Liu Y, Zhu Y, Jia W, Sun D, Zhao L, Zhang C, Wang C, Lyu Q, Chen Y, Chen G, Bo Y, and Xing Y. Association of the total white blood cell, neutrophils, and monocytes count with the presence, severity, and types of carotid atherosclerotic plaque. Front Med 7: 313, 2020. 10.3389/fmed.2020.00313 PMC7385072 32793608

[95] Ma L, Yang J, Runesha HB, Tanaka T, Ferrucci L, Bandinelli S, and Da Y. Genome-wide association analysis of total cholesterol and high-density lipoprotein cholesterol levels using the Framingham Heart Study data. BMC Med Genet 11: 55, 2010. 20370913 10.1186/1471-2350-11-55 PMC2867786

[96] Mackay F, Loetscher H, Stueber D, Gehr G, and Lesslauer W. Tumor necrosis factor alpha (TNF-alpha)-induced cell adhesion to human endothelial cells is under dominant control of one TNF receptor type, TNF-R55. J Exp Med 177: 1277–1286, 1993. 8386742 10.1084/jem.177.5.1277 PMC2190994

[97] Man JJ, Beckman JA, and Jaffe IZ. Sex as a biological variable in atherosclerosis. Circ Res 126: 1297–1319, 2020. 32324497 10.1161/CIRCRESAHA.120.315930 PMC7185045

[98] Martinez-Quinones P, McCarthy CG, Watts SW, Klee NS, Komic A, Calmasini FB, Priviero F, Warner A, Chenghao Y, and Wenceslau CF. Hypertension induced morphological and physiological changes in cells of the arterial wall. Am J Hypertens 31: 1067–1078, 2018. 29788246 10.1093/ajh/hpy083 PMC6132119

[99] Mateo T, Nabah YNA, Taha MA, Mata M, Cerdá-Nicolás M, Proudfoot AEI, Stahl RAK, Issekutz AC, Cortijo J, Morcillo EJ, Jose PJ, and Sanz M-J. Angiotensin II-induced mononuclear leukocyte interactions with arteriolar and venular endothelium are mediated by the release of different CC chemokines. J Immunol 176: 5577–5586, 2006. 16622027 10.4049/jimmunol.176.9.5577

[100] Mauersberger C, Schunkert H, and Sager HB. Inflammation-related risk loci in genome-wide association studies of coronary artery disease. Cells 10: 440, 2021. 33669721 10.3390/cells10020440 PMC7921935

[101] Mawhin M-A, Tilly P, Zirka G, Charles A-L, Slimani F, Vonesch J-L, Michel J-B, Bäck M, Norel X, and Fabre J-E. Neutrophils recruited by leukotriene B4 induce features of plaque destabilization during endotoxaemia. Cardiovasc Res 114: 1656–1666, 2018. 29800147 10.1093/cvr/cvy130

[102] McAlpine CS, Kiss MG, Rattik S, He S, Vassalli A, Valet C, Anzai A, Chan CT, Mindur JE, Kahles F, Poller WC, Frodermann V, Fenn AM, Gregory AF, Halle L, Iwamoto Y, Hoyer FF, Binder CJ, Libby P, Tafti M, Scammell TE, Nahrendorf M, and Swirski FK. Sleep modulates haematopoiesis and protects against atherosclerosis. Nature 566: 383–387, 2019. 30760925 10.1038/s41586-019-0948-2 PMC6442744

[103] Meisel SR, Dayan KI, Pauzner H, Chetboun I, Arbel Y, David D, and Kutz I. Effect of Iraqi missile war on incidence of acute myocardial infarction and sudden death in Israeli civilians. Lancet 338: 660–661, 1991. 1679475 10.1016/0140-6736(91)91234-l

[104] Miller ES, Apple CG, Kannan KB, Funk ZM, Plazas JM, Efron PA, and Mohr AM. Chronic stress induces persistent low-grade inflammation. Am J Surg 218: 677–683, 2019. 31378316 10.1016/j.amjsurg.2019.07.006 PMC6768696

[105] Miller MR and Newby DE. Air pollution and cardiovascular disease: car sick. Cardiovasc Res 116: 279–294, 2019. 10.1093/cvr/cvz228 31583404

[106] Mittleman MA and Mostofsky E. Physical, psychological and chemical triggers of acute cardiovascular events. Circulation 124: 346–354, 2011. 21768552 10.1161/CIRCULATIONAHA.110.968776 PMC3139921

[107] Moore KJ and Tabas I. Macrophages in the pathogenesis of atherosclerosis. Cell 145: 341–355, 2011. 21529710 10.1016/j.cell.2011.04.005 PMC3111065

[108] Mostofsky E, Penner EA, and Mittleman MA. Outbursts of anger as a trigger of acute cardiovascular events: a systematic review and meta-analysis. Eur Heart J 35: 1404–1410, 2014. 24591550 10.1093/eurheartj/ehu033 PMC4043318

[109] Münzel T, Daiber A, Steven S, Tran LP, Ullmann E, Kossmann S, Schmidt FP, Oelze M, Xia N, Li H, Pinto A, Wild P, Pies K, Schmidt ER, Rapp S, and Kröller-Schön S. Effects of noise on vascular function, oxidative stress, and inflammation: mechanistic insight from studies in mice. Eur Heart J 38: ehx081, 2017. 10.1093/eurheartj/ehx081 PMC5837459 28329261

[110] Münzel T, Gori T, Al-Kindi S, Deanfield J, Lelieveld J, Daiber A, and Rajagopalan S. Effects of gaseous and solid constituents of air pollution on endothelial function. Eur Heart J 39: 3543–3550, 2018. 30124840 10.1093/eurheartj/ehy481 PMC6174028

[111] Münzel T, Hahad O, Kuntic M, Keaney JF, Deanfield JE, and Daiber A. Effects of tobacco cigarettes, e-cigarettes, and waterpipe smoking on endothelial function and clinical outcomes. Eur Heart J 41: ehaa460, 2020. 10.1093/eurheartj/ehaa460 PMC7454514 32585699

[112] Münzel T, Miller MR, Sørensen M, Lelieveld J, Daiber A, and Rajagopalan S. Reduction of environmental pollutants for prevention of cardiovascular disease: it's time to act. Eur Heart J 41: ehaa745, 2020. 10.1093/eurheartj/ehaa745 PMC7672530 33141181

[113] Münzel T, Sørensen M, Gori T, Schmidt FP, Rao X, Brook FR, Chen LC, Brook RD, and Rajagopalan S. Environmental stressors and cardio-metabolic disease: part II–mechanistic insights. Eur Heart J 38: 557–564, 2017. 27460891 10.1093/eurheartj/ehw294 PMC5381593

[114] Musher DM, Abers MS, and Corrales-Medina VF. Acute infection and myocardial infarction. N Engl J Med 380: 171–176, 2019. 30625066 10.1056/NEJMra1808137

[115] Nabi H, Kivimäki M, Batty GD, Shipley MJ, Britton A, Brunner EJ, Vahtera J, Lemogne C, Elbaz A, and Singh-Manoux A. Increased risk of coronary heart disease among individuals reporting adverse impact of stress on their health: the Whitehall II prospective cohort study. Eur Heart J 34: 2697–2705, 2013. 23804585 10.1093/eurheartj/eht216 PMC3766148

[116] Nahrendorf M. Multiorgan imaging of comorbidity and cardiovascular risk. JACC Cardiovasc Imaging 13: 478–480, 2020. 30448129 10.1016/j.jcmg.2018.09.017

[117] Nahrendorf M and Swirski FK. Lifestyle effects on hematopoiesis and atherosclerosis. Circ Res 116: 884–894, 2015. 25722442 10.1161/CIRCRESAHA.116.303550 PMC4347940

[118] Najafi AH, Aghili N, Tilan JU, Andrews JA, Peng X, Lassance-Soares RM, Sood S, Alderman LO, Abe K, Li L, Kolodgie FD, Virmani R, Zukowska Z, Epstein SE, and Burnett MS. A new murine model of stress-induced complex atherosclerotic lesions. Dis Model Mech 6: 323–331, 2013. 23324329 10.1242/dmm.009977 PMC3597015

[119] Netea MG, Domínguez-Andrés J, Barreiro LB, Chavakis T, Divangahi M, Fuchs E, Joosten LAB, Meer JWM van der, Mhlanga MM, Mulder WJM, Riksen NP, Schlitzer A, Schultze JL, Benn CS, Sun JC, Xavier RJ, and Latz E. Defining trained immunity and its role in health and disease. Nat Rev Immunol 20: 375–388, 2020. 32132681 10.1038/s41577-020-0285-6 PMC7186935

[120] Nollet M, Guisquet AL, and Belzung C. Models of depression: unpredictable chronic mild stress in mice. Curr Protoc Pharmacol 61: 5.65.1–5.65.17, 2013. 10.1002/0471141755.ph0565s61 23744712

[121] Ortega FB, Lavie CJ, and Blair SN. Obesity and cardiovascular disease. Circ Res 118: 1752–1770, 2016. 27230640 10.1161/CIRCRESAHA.115.306883

[122] Osborne MT, Shin LM, Mehta NN, Pitman RK, Fayad ZA, and Tawakol A. Disentangling the links between psychosocial stress and cardiovascular disease. Circ Cardiovasc Imaging 13: e010931, 2020. 32791843 10.1161/CIRCIMAGING.120.010931 PMC7430065

[123] Pennathur S and Heinecke JW. Mechanisms for oxidative stress in diabetic cardiovascular disease. Antioxid Redox Signal 9: 955–969, 2007. 17508917 10.1089/ars.2007.1595

[124] Pierce H, Zhang D, Magnon C, Lucas D, Christin JR, Huggins M, Schwartz GJ, and Frenette PS. Cholinergic signals from the CNS regulate G-CSF-mediated HSC mobilization from bone marrow via a glucocorticoid signaling relay. Cell Stem Cell 20: 648.e4–658.e4, 2017. 28196601 10.1016/j.stem.2017.01.002 PMC5467872

[125] Poitras VJ and Pyke KE. The impact of acute mental stress on vascular endothelial function: evidence, mechanisms and importance. Int J Psychophysiol 88: 124–135, 2013. 23562766 10.1016/j.ijpsycho.2013.03.019

[126] Powell ND, Sloan EK, Bailey MT, Arevalo JMG, Miller GE, Chen E, Kobor MS, Reader BF, Sheridan JF, and Cole SW. Social stress up-regulates inflammatory gene expression in the leukocyte transcriptome via β-adrenergic induction of myelopoiesis. Proc Natl Acad Sci U S A 110: 16574–16579, 2013. 24062448 10.1073/pnas.1310655110 PMC3799381

[127] Puhlmann M, Weinreich DM, Farma JM, Carroll NM, Turner EM, and Alexander HR. Interleukin-1β induced vascular permeability is dependent on induction of endothelial Tissue Factor (TF) activity. J Transl Med 3: 37, 2005. 16197553 10.1186/1479-5876-3-37 PMC1276820

[128] Qing H, Desrouleaux R, Israni-Winger K, Mineur YS, Fogelman N, Zhang C, Rashed S, Palm NW, Sinha R, Picciotto MR, Perry RJ, and Wang A. Origin and function of stress-induced IL-6 in murine models. Cell 182: 372.e14–387.e14, 2020. 32610084 10.1016/j.cell.2020.05.054 PMC7384974

[129] Randolph GJ. Mechanisms that regulate macrophage burden in atherosclerosis. Circ Res 114: 1757–1771, 2014. 24855200 10.1161/CIRCRESAHA.114.301174 PMC4059102

[130] Recio-Mayoral A, Mason JC, Kaski JC, Rubens MB, Harari OA, and Camici PG. Chronic inflammation and coronary microvascular dysfunction in patients without risk factors for coronary artery disease. Eur Heart J 30: 1837–1843, 2009. 19502228 10.1093/eurheartj/ehp205

[131] Recio-Mayoral A, Rimoldi OE, Camici PG, and Kaski JC. Inflammation and microvascular dysfunction in cardiac syndrome X patients without conventional risk factors for coronary artery disease. JACC Cardiovasc Imaging 6: 660–667, 2013. 23643286 10.1016/j.jcmg.2012.12.011

[132] Ridker PM. Residual inflammatory risk: addressing the obverse side of the atherosclerosis prevention coin. Eur Heart J 37: 1720–1722, 2016. 26908943 10.1093/eurheartj/ehw024

[133] Ridker PM, Everett BM, Thuren T, MacFadyen JG, Chang WH, Ballantyne C, Fonseca F, Nicolau J, Koenig W, Anker SD, Kastelein JJP, Cornel JH, Pais P, Pella D, Genest J, Cifkova R, Lorenzatti A, Forster T, Kobalava Z, Vida-Simiti L, Flather M, Shimokawa H, Ogawa H, Dellborg M, Rossi PRF, Troquay RPT, Libby P, and Glynn RJ; CANTOS Trial Group. Antiinflammatory therapy with canakinumab for atherosclerotic disease. N Engl J Med 377: 1119–1131, 2017. 28845751 10.1056/NEJMoa1707914

[134] Ridker PM, MacFadyen JG, Glynn RJ, Bradwin G, Hasan AA, and Rifai N. Comparison of interleukin-6, C-reactive protein, and low-density lipoprotein cholesterol as biomarkers of residual risk in contemporary practice: secondary analyses from the Cardiovascular Inflammation Reduction Trial. Eur Heart J 41: 2952–2961, 2020. 32221587 10.1093/eurheartj/ehaa160 PMC7453833

[135] Robbins CS, Hilgendorf I, Weber GF, Theurl I, Iwamoto Y, Figueiredo J-L, Gorbatov R, Sukhova GK, Gerhardt LMS, Smyth D, Zavitz CCJ, Shikatani EA, Parsons M, Rooijen N van, Lin HY, Husain M, Libby P, Nahrendorf M, Weissleder R, and Swirski FK. Local proliferation dominates lesional macrophage accumulation in atherosclerosis. Nat Med 19: 1166–1172, 2013. 23933982 10.1038/nm.3258 PMC3769444

[136] Roth GA, Mensah GA, Johnson CO, Addolorato G, Ammirati E, Baddour LM, Barengo NC, Beaton AZ, Benjamin EJ, Benziger CP, Bonny A, Brauer M, Brodmann M, Cahill TJ, Carapetis J, Catapano AL, Chugh SS, Cooper LT, Coresh J, Criqui M, DeCleene N, Eagle KA, Emmons-Bell S, Feigin VL, Fernández-Solà J, Fowkes G, Gakidou E, Grundy SM, He FJ, Howard G, Hu F, Inker L, Karthikeyan G, Kassebaum N, Koroshetz W, Lavie C, Lloyd-Jones D, Lu HS, Mirijello A, Temesgen AM, Mokdad A, Moran AE, Muntner P, Narula J, Neal B, Ntsekhe M, de Oliveira GM, Otto C, Owolabi M, Pratt M, Rajagopalan S, Reitsma M, Ribeiro ALP, Rigotti N, Rodgers A, Sable C, Shakil S, Sliwa-Hahnle K, Stark B, Sundström J, Timpel P, Tleyjeh IM, Valgimigli M, Vos T, Whelton PK, Yacoub M, Zuhlke L, Murray C, Fuster V; GBD-NHLBI-JACC Global Burden of Cardiovascular Diseases Writing Group; Roth GA, Mensah GA, Johnson CO, Addolorato G, Ammirati E, Baddour LM, Barengo NC, Beaton A, Benjamin EJ, Benziger CP, Bonny A, Brauer M, Brodmann M, Cahill TJ, Carapetis JR, Catapano AL, Chugh S, Cooper LT, Coresh J, Criqui MH, DeCleene NK, Eagle KA, Emmons-Bell S, Feigin VL, Fernández-Sola J, Fowkes FGR, Gakidou E, Grundy SM, He FJ, Howard G, Hu F, Inker L, Karthikeyan G, Kassebaum NJ, Koroshetz WJ, Lavie C, Lloyd-Jones D, Lu HS, Mirijello A, Misganaw AT, Mokdad AH, Moran AE, Muntner P, Narula J, Neal B, Ntsekhe M, Oliveira GMM, Otto CM, Owolabi MO, Pratt M, Rajagopalan S, Reitsma MB, Ribeiro ALP, Rigotti NA, Rodgers A, Sable CA, Shakil SS, Sliwa K, Stark BA, Sundström J, Timpel P, Tleyjeh II, Valgimigli M, Vos T, Whelton PK, Yacoub M, Zuhlke LJ, Abbasi-Kangevari M, Abdi A, Abedi A, Aboyans V, Abrha WA, Abu-Gharbieh E, Abushouk AI, Acharya D, Adair T, Adebayo OM, Ademi Z, Advani SM, Afshari K, Afshin A, Agarwal G, Agasthi P, Ahmad S, Ahmadi S, Ahmed MB, Aji B, Akalu Y, Akande-Sholabi W, Aklilu A, Akunna CJ, Alahdab F, Al-Eyadhy A, Alhabib KF, Alif SM, Alipour V, Aljunid SM, Alla F, Almasi-Hashiani A, Almustanyir S, Al-Raddadi RM, Amegah AK, Amini S, Aminorroaya A, Amu H, Amugsi DA, Ancuceanu R, Anderlini D, Andrei T, Andrei CL, Ansari-Moghaddam A, Anteneh ZA, Antonazzo IC, Antony B, Anwer R, Appiah LT, Arabloo J, Ärnlöv J, Artanti KD, Ataro Z, Ausloos M, Avila-Burgos L, Awan AT, Awoke MA, Ayele HT, Ayza MA, Azari S, B DB, Baheiraei N, Baig AA, Bakhtiari A, Banach M, Banik PC, Baptista EA, Barboza MA, Barua L, Basu S, Bedi N, Béjot Y, Bennett DA, Bensenor IM, Berman AE, Bezabih YM, Bhagavathula AS, Bhaskar S, Bhattacharyya K, Bijani A, Bikbov B, Birhanu MM, Boloor A, Brant LC, Brenner H, Briko NI, Butt ZA, Santos FLC dos, Cahill LE, Cahuana-Hurtado L, Cámera LA, Campos-Nonato IR, Cantu-Brito C, Car J, Carrero JJ, Carvalho F, Castañeda-Orjuela CA, Catalá-López F, Cerin E, Charan J, Chattu VK, Chen S, Chin KL, Choi J-YJ, Chu D-T, Chung S-C, Cirillo M, Coffey S, Conti S, Costa VM, Cundiff DK, Dadras O, Dagnew B, Dai X, Damasceno AAM, Dandona L, Dandona R, Davletov K, Cruz-Góngora VD la, Hoz FPD la, Neve J-WD, Denova-Gutiérrez E, Molla MD, Derseh BT, Desai R, Deuschl G, Dharmaratne SD, Dhimal M, Dhungana RR, Dianatinasab M, Diaz D, Djalalinia S, Dokova K, Douiri A, Duncan BB, Duraes AR, Eagan AW, Ebtehaj S, Eftekhari A, Eftekharzadeh S, Ekholuenetale M, Nahas NE, Elgendy IY, Elhadi M, El-Jaafary SI, Esteghamati S, Etisso AE, Eyawo O, Fadhil I, Faraon EJA, Faris PS, Farwati M, Farzadfar F, Fernandes E, Prendes CF, Ferrara P, Filip I, Fischer F, Flood D, Fukumoto T, Gad MM, Gaidhane S, Ganji M, Garg J, Gebre AK, Gebregiorgis BG, Gebregzabiher KZ, Gebremeskel GG, Getacher L, Obsa AG, Ghajar A, Ghashghaee A, Ghith N, Giampaoli S, Gilani SA, Gill PS, Gillum RF, Glushkova EV, Gnedovskaya EV, Golechha M, Gonfa KB, Goudarzian AH, Goulart AC, Guadamuz JS, Guha A, Guo Y, Gupta R, Hachinski V, Hafezi-Nejad N, Haile TG, Hamadeh RR, Hamidi S, Hankey GJ, Hargono A, Hartono RK, Hashemian M, Hashi A, Hassan S, Hassen HY, Havmoeller RJ, Hay SI, Hayat K, Heidari G, Herteliu C, Holla R, Hosseini M, Hosseinzadeh M, Hostiuc M, Hostiuc S, Househ M, Huang J, Humayun A, Iavicoli I, Ibeneme CU, Ibitoye SE, Ilesanmi OS, Ilic IM, Ilic MD, Iqbal U, Irvani SSN, Islam SMS, Islam RM, Iso H, Iwagami M, Jain V, Javaheri T, Jayapal SK, Jayaram S, Jayawardena R, Jeemon P, Jha RP, Jonas JB, Jonnagaddala J, Joukar F, Jozwiak JJ, Jürisson M, Kabir A, Kahlon T, Kalani R, Kalhor R, Kamath A, Kamel I, Kandel H, Kandel A, Karch A, Kasa AS, Katoto PDMC, Kayode GA, Khader YS, Khammarnia M, Khan MS, Khan MN, Khan M, Khan EA, Khatab K, Kibria GMA, Kim YJ, Kim GR, Kimokoti RW, Kisa S, Kisa A, Kivimäki M, Kolte D, Koolivand A, Korshunov VA, Laxminarayana SLK, Koyanagi A, Krishan K, Krishnamoorthy V, Defo BK, Bicer BK, Kulkarni V, Kumar GA, Kumar N, Kurmi OP, Kusuma D, Kwan GF, Vecchia CL, Lacey B, Lallukka T, Lan Q, Lasrado S, Lassi ZS, Lauriola P, Lawrence WR, Laxmaiah A, LeGrand KE, Li M-C, Li B, Li S, Lim SS, Lim L-L, Lin H, Lin Z, Lin R-T, Liu X, Lopez AD, Lorkowski S, Lotufo PA, Lugo A, M NK, Madotto F, Mahmoudi M, Majeed A, Malekzadeh R, Malik AA, Mamun AA, Manafi N, Mansournia MA, Mantovani LG, Martini S, Mathur MR, Mazzaglia G, Mehata S, Mehndiratta MM, Meier T, Menezes RG, Meretoja A, Mestrovic T, Miazgowski B, Miazgowski T, Michalek IM, Miller TR, Mirrakhimov EM, Mirzaei H, Moazen B, Moghadaszadeh M, Mohammad Y, Mohammad DK, Mohammed S, Mohammed MA, Mokhayeri Y, Molokhia M, Montasir AA, Moradi G, Moradzadeh R, Moraga P, Morawska L, Velásquez IM, Morze J, Mubarik S, Muruet W, Musa KI, Nagarajan AJ, Nalini M, Nangia V, Naqvi AA, Swamy SN, Nascimento BR, Nayak VC, Nazari J, Nazarzadeh M, Negoi RI, Kandel SN, Nguyen HLT, Nixon MR, Norrving B, Noubiap JJ, Nouthe BE, Nowak C, Odukoya OO, Ogbo FA, Olagunju AT, Orru H, Ortiz A, Ostroff SM, Padubidri JR, Palladino R, Pana A, Panda-Jonas S, Parekh U, Park E-C, Parvizi M, Kan FP, Patel UK, Pathak M, Paudel R, Pepito VCF, Perianayagam A, Perico N, Pham HQ, Pilgrim T, Piradov MA, Pishgar F, Podder V, Polibin RV, Pourshams A, Pribadi DRA, Rabiee N, Rabiee M, Radfar A, Rafiei A, Rahim F, Rahimi-Movaghar V, Rahman MHU, Rahman MA, Rahmani AM, Rakovac I, Ram P, Ramalingam S, Rana J, Ranasinghe P, Rao SJ, Rathi P, Rawal L, Rawasia WF, Rawassizadeh R, Remuzzi G, Renzaho AMN, Rezapour A, Riahi SM, Roberts-Thomson RL, Roever L, Rohloff P, Romoli M, Roshandel G, Rwegerera GM, Saadatagah S, Saber-Ayad MM, Sabour S, Sacco S, Sadeghi M, Moghaddam SS, Safari S, Sahebkar A, Salehi S, Salimzadeh H, Samaei M, Samy AM, Santos IS, Santric-Milicevic MM, Sarrafzadegan N, Sarveazad A, Sathish T, Sawhney M, Saylan M, Schmidt MI, Schutte AE, Senthilkumaran S, Sepanlou SG, Sha F, Shahabi S, Shahid I, Shaikh MA, Shamali M, Shamsizadeh M, Shawon MSR, Sheikh A, Shigematsu M, Shin M-J, Shin JI, Shiri R, Shiue I, Shuval K, Siabani S, Siddiqi TJ, Silva DAS, Singh JA, Mtech AS, Skryabin VY, Skryabina AA, Soheili A, Spurlock EE, Stockfelt L, Stortecky S, Stranges S, Abdulkader RS, Tadbiri H, Tadesse EG, Tadesse DB, Tajdini M, Tariqujjaman M, Teklehaimanot BF, Temsah M-H, Tesema AK, Thakur B, Thankappan KR, Thapar R, Thrift AG, Timalsina B, Tonelli M, Touvier M, Tovani-Palone MR, Tripathi A, Tripathy JP, Truelsen TC, Tsegay GM, Tsegaye GW, Tsilimparis N, Tusa BS, Tyrovolas S, Umapathi KK, Unim B, Unnikrishnan B, Usman MS, Vaduganathan M, Valdez PR, Vasankari TJ, Velazquez DZ, Venketasubramanian N, Vu GT, Vujcic IS, Waheed Y, Wang Y, Wang F, Wei J, Weintraub RG, Weldemariam AH, Westerman R, Winkler AS, Wiysonge CS, Wolfe CDA, Wubishet BL, Xu G, Yadollahpour A, Yamagishi K, Yan LL, Yandrapalli S, Yano Y, Yatsuya H, Yeheyis TY, Yeshaw Y, Yilgwan CS, Yonemoto N, Yu C, Yusefzadeh H, Zachariah G, Zaman SB, Zaman MS, Zamanian M, Zand R, Zandifar A, Zarghi A, Zastrozhin MS, Zastrozhina A, Zhang Z-J, Zhang Y, Zhang W, Zhong C, Zou Z, Zuniga YMH, Murray CJL, and Fuster V. Global burden of cardiovascular diseases and risk factors, 1990–2019 update from the GBD 2019 study. J Am Coll Cardiol 76: 2982–3021, 2020. 33309175 10.1016/j.jacc.2020.11.010 PMC7755038

[137] Roth L, Rombouts M, Schrijvers DM, Lemmens K, Keulenaer GWD, Martinet W, and Meyer GRYD. Chronic intermittent mental stress promotes atherosclerotic plaque vulnerability, myocardial infarction and sudden death in mice. Atherosclerosis 242: 288–294, 2015. 26233915 10.1016/j.atherosclerosis.2015.07.025

[138] Rozanski A, Blumenthal JA, and Kaplan J. Impact of psychological factors on the pathogenesis of cardiovascular disease and implications for therapy. Circulation 99: 2192–2217, 1999. 10217662 10.1161/01.cir.99.16.2192

[139] Sager HB, Dutta P, Dahlman JE, Hulsmans M, Courties G, Sun Y, Heidt T, Vinegoni C, Borodovsky A, Fitzgerald K, Wojtkiewicz GR, Iwamoto Y, Tricot B, Khan OF, Kauffman KJ, Xing Y, Shaw TE, Libby P, Langer R, Weissleder R, Swirski FK, Anderson DG, and Nahrendorf M. RNAi targeting multiple cell adhesion molecules reduces immune cell recruitment and vascular inflammation after myocardial infarction. Sci Transl Med 8: 342ra80, 2016. 10.1126/scitranslmed.aaf1435 PMC5125383 27280687

[140] Sager HB and Koenig W. Acute inflammation and long-term cardiovascular risk: identifying an unrecognised vulnerable gap. Eur J Prev Cardiol 24: 1956–1957, 2017. 29048207 10.1177/2047487317736869

[141] Sager HB and Nahrendorf M. Inflammation: a trigger for acute coronary syndrome. Q J Nucl Med Mol Imaging 60: 185–193, 2016. 27273431

[142] Salvador AF, de Lima KA, and Kipnis J. Neuromodulation by the immune system: a focus on cytokines. Nat Rev Immunol 21: 526–541, 2021. 33649606 10.1038/s41577-021-00508-z

[143] Scally C, Abbas H, Ahearn T, Srinivasan J, Mezincescu A, Rudd A, Spath N, Yucel-Finn A, Yuecel R, Oldroyd K, Dospinescu C, Horgan G, Broadhurst P, Henning A, Newby DE, Semple S, Wilson H, and Dawson DK. Myocardial and systemic inflammation in acute stress-induced (takotsubo) cardiomyopathy. Circulation 139: 1581–1592, 2019. 30586731 10.1161/CIRCULATIONAHA.118.037975 PMC6438459

[144] Schakel L, Veldhuijzen DS, Crompvoets PI, Bosch JA, Cohen S, van Middendorp H, Joosten SA, Ottenhoff THM, Visser LG, and Evers AWM. Effectiveness of stress-reducing interventions on the response to challenges to the immune system: a meta-analytic review. Psychother Psychosom 88: 274–286, 2019. 31387109 10.1159/000501645 PMC6878733

[145] Scheiermann C, Kunisaki Y, Lucas D, Chow A, Zhang JE, Zhang D, Hashimoto DMM, and Frenette PS. Adrenergic nerves govern circadian leukocyte recruitment to tissues. Immunity 37: 290–301, 2012. 22863835 10.1016/j.immuni.2012.05.021 PMC3428436

[146] Schloss MJ, Swirski FK, and Nahrendorf M. Modifiable cardiovascular risk, hematopoiesis, and innate immunity. Circ Res 126: 1242–1259, 2020. 32324501 10.1161/CIRCRESAHA.120.315936 PMC7185037

[147] Schweizer MC, Henniger MSH, and Sillaber I. Chronic mild stress (CMS) in mice: of anhedonia, ‘anomalous anxiolysis' and activity. PLoS One 4: e4326, 2009. 19177164 10.1371/journal.pone.0004326 PMC2627902

[148] Sher LD, Geddie H, Olivier L, Cairns M, Truter N, Beselaar L, and Essop MF. Chronic stress and endothelial dysfunction: mechanisms, experimental challenges, and the way ahead. Am J Physiol Heart Circ Physiol 319: H488–H506, 2020. 32618516 10.1152/ajpheart.00244.2020

[149] Siggins RW, Hossain F, Rehman T, Melvan JN, Zhang P, and Welsh DA. Cigarette smoke alters the hematopoietic stem cell niche. Med Sci (Basel) 2: 37–50, 2014. 28868162 10.3390/medsci2010037 PMC5576506

[150] Silvestre-Roig C, Braster Q, Ortega-Gomez A, and Soehnlein O. Neutrophils as regulators of cardiovascular inflammation. Nat Rev Cardiol 17: 327–340, 2020. 31996800 10.1038/s41569-019-0326-7

[151] Silvestre-Roig C, Braster Q, Wichapong K, Lee EY, Teulon JM, Berrebeh N, Winter J, Adrover JM, Santos GS, Froese A, Lemnitzer P, Ortega-Gómez A, Chevre R, Marschner J, Schumski A, Winter C, Perez-Olivares L, Pan C, Paulin N, Schoufour T, Hartwig H, González-Ramos S, Kamp F, Megens RTA, Mowen KA, Gunzer M, Maegdefessel L, Hackeng T, Lutgens E, Daemen M, Blume J von, Anders H-J, Nikolaev VO, Pellequer J-L, Weber C, Hidalgo A, Nicolaes GAF, Wong GCL, and Soehnlein O. Externalized histone H4 orchestrates chronic inflammation by inducing lytic cell death. Nature 569: 236–240, 2019. 31043745 10.1038/s41586-019-1167-6 PMC6716525

[152] Silvestre-Roig C, de Winther MP, Weber C, Daemen MJ, Lutgens E, and Soehnlein O. Atherosclerotic plaque destabilization. Circ Res 114: 214–226, 2014. 24385514 10.1161/CIRCRESAHA.114.302355

[153] Skeoch S and Bruce IN. Atherosclerosis in rheumatoid arthritis: is it all about inflammation? Nat Rev Rheumatol 11: 390–400, 2015. 25825281 10.1038/nrrheum.2015.40

[154] Smith CJ, Kluck LA, Ruan GJ, Ashrani AA, Marshall AL, Pruthi RK, Shah MV, Wolanskyj-Spinner A, Gangat N, Litzow MR, Hogan WJ, Sridharan M, and Go RS. Leukocytosis and tobacco use: an observational study of asymptomatic leukocytosis. Am J Med 134: e31–e35, 2020. 32682870 10.1016/j.amjmed.2020.06.014

[155] Smyth A, O'Donnell M, Lamelas P, Teo K, Rangarajan S, Yusuf S, and Investigators I. Physical activity and anger or emotional upset as triggers of acute myocardial infarction. Circulation 134: 1059–1067, 2016. 27753614 10.1161/CIRCULATIONAHA.116.023142

[156] Smyth GP, Stapleton PP, Freeman TA, Concannon EM, Mestre JR, Duff M, Maddali S, and Daly JM. Glucocorticoid pretreatment induces cytokine overexpression and nuclear factor-κB activation in macrophages. J Surg Res 116: 253–261, 2004. 15013364 10.1016/S0022-4804(03)00300-7

[157] Snijders C, Pries L-K, Sgammeglia N, Jowf GA, Youssef NA, de Nijs L, Guloksuz S, and Rutten BPF. Resilience against traumatic stress: current developments and future directions. Front Psychiatry 9: 676, 2018. 30631285 10.3389/fpsyt.2018.00676 PMC6315131

[158] Soehnlein O. Multiple roles for neutrophils in atherosclerosis. Circ Res 110: 875–888, 2012. 22427325 10.1161/CIRCRESAHA.111.257535

[159] Steptoe A and Kivimäki M. Stress and cardiovascular disease. Nat Rev Cardiol 9: 360–370, 2012. 22473079 10.1038/nrcardio.2012.45

[160] Strike PC, Magid K, Whitehead DL, Brydon L, Bhattacharyya MR, and Steptoe A. Pathophysiological processes underlying emotional triggering of acute cardiac events. Proc Natl Acad Sci U S A 103: 4322–4327, 2006. 16537529 10.1073/pnas.0507097103 PMC1449691

[161] Tabas I, García-Cardeña G, and Owens GK. Recent insights into the cellular biology of atherosclerosisThe cellular biology of atherosclerosis. J Cell Biol 209: 13–22, 2015. 25869663 10.1083/jcb.201412052 PMC4395483

[162] Tall AR and Jelic S. How broken sleep promotes cardiovascular disease. Nature 566: 329–330, 2019. 30783270 10.1038/d41586-019-00393-6

[163] Tardif J-C, Kouz S, Waters DD, Bertrand OF, Diaz R, Maggioni AP, Pinto FJ, Ibrahim R, Gamra H, Kiwan GS, Berry C, López-Sendón J, Ostadal P, Koenig W, Angoulvant D, Grégoire JC, Lavoie M-A, Dubé M-P, Rhainds D, Provencher M, Blondeau L, Orfanos A, L'Allier PL, Guertin M-C, and Roubille F. Efficacy and safety of low-dose colchicine after myocardial infarction. N Engl J Med 381: 2497–2505, 2019. 31733140 10.1056/NEJMoa1912388

[164] Tawakol A, Ishai A, Takx RA, Figueroa AL, Ali A, Kaiser Y, Truong QA, Solomon CJ, Calcagno C, Mani V, Tang CY, Mulder WJ, Murrough JW, Hoffmann U, Nahrendorf M, Shin LM, Fayad ZA, and Pitman RK. Relation between resting amygdalar activity and cardiovascular events: a longitudinal and cohort study. Lancet 389: 834–845, 2017. 28088338 10.1016/S0140-6736(16)31714-7 PMC7864285

[165] Tobaldini E, Fiorelli EM, Solbiati M, Costantino G, Nobili L, and Montano N. Short sleep duration and cardiometabolic risk: from pathophysiology to clinical evidence. Nat Rev Cardiol 16: 213–224, 2019. 30410106 10.1038/s41569-018-0109-6

[166] Tsou C-L, Peters W, Si Y, Slaymaker S, Aslanian AM, Weisberg SP, Mack M, and Charo IF. Critical roles for CCR2 and MCP-3 in monocyte mobilization from bone marrow and recruitment to inflammatory sites. J Clin Invest 117: 902–909, 2007. 17364026 10.1172/JCI29919 PMC1810572

[167] Tyrrell DJ and Goldstein DR. Ageing and atherosclerosis: vascular intrinsic and extrinsic factors and potential role of IL-6. Nat Rev Cardiol 18: 58–68, 2021. 32918047 10.1038/s41569-020-0431-7 PMC7484613

[168] Vaccarino V, Sullivan S, Hammadah M, Wilmot K, Mheid IA, Ramadan R, Elon L, Pimple PM, Garcia EV, Nye J, Shah AJ, Alkhoder A, Levantsevych O, Gay H, Obideen M, Huang M, Lewis TT, Bremner JD, Quyyumi AA, and Raggi P. Mental stress–induced-myocardial ischemia in young patients with recent myocardial infarction. Circulation 137: 794–805, 2018. 29459465 10.1161/CIRCULATIONAHA.117.030849 PMC5822741

[169] Vasamsetti SB, Florentin J, Coppin E, Stiekema LCA, Zheng KH, Nisar MU, Sembrat J, Levinthal DJ, Rojas M, Stroes ESG, Kim K, and Dutta P. Sympathetic neuronal activation triggers myeloid progenitor proliferation and differentiation. Immunity 49: 93.e7–106.e7, 2018. 29958804 10.1016/j.immuni.2018.05.004 PMC6051926

[170] Vergallo R and Crea F. Atherosclerotic plaque healing. N Engl J Med 383: 846–857, 2020. 32846063 10.1056/NEJMra2000317

[171] Vermeulen R, Schymanski EL, Barabási A-L, and Miller GW. The exposome and health: where chemistry meets biology. Science 367: 392–396, 2020. 31974245 10.1126/science.aay3164 PMC7227413

[172] Vickers K, Jafarpour S, Mofidi A, Rafat B, and Woznica A. The 35% carbon dioxide test in stress and panic research: overview of effects and integration of findings. Clin Psychol Rev 32: 153–164, 2012. 22322014 10.1016/j.cpr.2011.12.004

[173] Vineis P, Chadeau-Hyam M, Gmuender H, Gulliver J, Herceg Z, Kleinjans J, Kogevinas M, Kyrtopoulos S, Nieuwenhuijsen M, Phillips DH, Probst-Hensch N, Scalbert A, Vermeulen R, and Wild CP; EXPOsOMICS Consortium. The exposome in practice: design of the EXPOsOMICS project. Int J Hyg Environ Health 220: 142–151, 2017. 27576363 10.1016/j.ijheh.2016.08.001 PMC6192011

[174] Virtanen M and Kivimäki M. Long working hours and risk of cardiovascular disease. Curr Cardiol Rep 20: 123, 2018. 30276493 10.1007/s11886-018-1049-9 PMC6267375

[175] Viswanathan K and Dhabhar FS. Stress-induced enhancement of leukocyte trafficking into sites of surgery or immune activation. Proc Natl Acad Sci U S A 102: 5808–5813, 2005. 15817686 10.1073/pnas.0501650102 PMC556309

[176] Vromman A, Ruvkun V, Shvartz E, Wojtkiewicz G, Masson GS, Tesmenitsky Y, Folco E, Gram H, Nahrendorf M, Swirski FK, Sukhova GK, and Libby P. Stage-dependent differential effects of interleukin-1 isoforms on experimental atherosclerosis. Eur Heart J 40: 2482–2491, 2019. 30698710 10.1093/eurheartj/ehz008 PMC6685323

[177] Wang JC and Bennett M. Aging and atherosclerosis. Circ Res 111: 245–259, 2012. 22773427 10.1161/CIRCRESAHA.111.261388

[178] Wang Z, Bergeron N, Levison BS, Li XS, Chiu S, Jia X, Koeth RA, Li L, Wu Y, Tang WHW, Krauss RM, and Hazen SL. Impact of chronic dietary red meat, white meat, or non-meat protein on trimethylamine N-oxide metabolism and renal excretion in healthy men and women. Eur Heart J 40: 583–594, 2018. 10.1093/eurheartj/ehy799 PMC6374688 30535398

[179] Westphal M, Bingisser M-B, Feng T, Wall M, Blakley E, Bingisser R, and Kleim B. Protective benefits of mindfulness in emergency room personnel. J Affect Disord 175: 79–85, 2015. 25597793 10.1016/j.jad.2014.12.038

[180] Wieduwild E, Girard-Madoux MJ, Quatrini L, Laprie C, Chasson L, Rossignol R, Bernat C, Guia S, and Ugolini S. β2-adrenergic signals downregulate the innate immune response and reduce host resistance to viral infection. J Exp Med 217: e20190554, 2020. 32045472 10.1084/jem.20190554 PMC7144531

[181] Wilbert-Lampen U, Leistner D, Greven S, Pohl T, Sper S, Völker C, Güthlin D, Plasse A, Knez A, Küchenhoff H, and Steinbeck G. Cardiovascular events during World Cup soccer. N Engl J Med 358: 475–483, 2008. 18234752 10.1056/NEJMoa0707427

[182] Wilbert-Lampen U, Nickel T, Leistner D, Güthlin D, Matis T, Völker C, Sper S, Küchenhoff H, Kääb S, and Steinbeck G. Modified serum profiles of inflammatory and vasoconstrictive factors in patients with emotional stress-induced acute coronary syndrome during World Cup soccer 2006. J Am Coll Cardiol 55: 637–642, 2010. 20170788 10.1016/j.jacc.2009.07.073

[183] Winkels H, Ehinger E, Vassallo M, Buscher K, Dinh HQ, Kobiyama K, Hamers AAJ, Cochain C, Vafadarnejad E, Saliba A-E, Zernecke A, Pramod AB, Ghosh AK, Michel NA, Hoppe N, Hilgendorf I, Zirlik A, Hedrick CC, Ley K, and Wolf D. Atlas of the immune cell repertoire in mouse atherosclerosis defined by single-cell RNA-sequencing and mass cytometry. Circ Res 122: 1675–1688, 2018. 29545366 10.1161/CIRCRESAHA.117.312513 PMC5993603

[184] Wolf D and Ley K. Immunity and inflammation in atherosclerosis. Circ Res 124: 315–327, 2019. 30653442 10.1161/CIRCRESAHA.118.313591 PMC6342482

[185] Wood SK and Bhatnagar S. Resilience to the effects of social stress: evidence from clinical and preclinical studies on the role of coping strategies. Neurobiol Stress 1: 164–173, 2015. 25580450 10.1016/j.ynstr.2014.11.002 PMC4286805

[186] Wouw M van de, Lyte JM, Boehme M, Sichetti M, Moloney G, Goodson MS, Kelley-Loughnane N, Dinan TG, Clarke G, and Cryan JF. The role of the microbiota in acute stress-induced myeloid immune cell trafficking. Brain Behav Immun 84: 209–217, 2020. 31812778 10.1016/j.bbi.2019.12.003

[187] Xu C, Lee SK, Zhang D, and Frenette PS. The gut microbiome regulates psychological-stress-induced inflammation. Immunity 53: 417.e4–428.e4, 2020. 32735844 10.1016/j.immuni.2020.06.025 PMC7461158

[188] Yusuf S, Hawken S, Ôunpuu S, Dans T, Avezum A, Lanas F, McQueen M, Budaj A, Pais P, Varigos J, and Lisheng L; INTERHEART Study Investigators. Effect of potentially modifiable risk factors associated with myocardial infarction in 52 countries (the INTERHEART study): case-control study. Lancet 364: 937–952, 2004. 15364185 10.1016/S0140-6736(04)17018-9

[189] Yvan-Charvet L, Pagler T, Gautier EL, Avagyan S, Siry RL, Han S, Welch CL, Wang N, Randolph GJ, Snoeck HW, and Tall AR. ATP-binding cassette transporters and HDL suppress hematopoietic stem cell proliferation. Science 328: 1689–1693, 2010. 20488992 10.1126/science.1189731 PMC3032591

[190] Zernecke A and Weber C. Chemokines in atherosclerosis. Arterioscler Thromb Vasc Biol 34: 742–750, 2014. 24436368 10.1161/ATVBAHA.113.301655

[191] Zhang T, Zhai Y, Chen Y, Zhou Z, Yang J, and Liu H. Effects of emotional and physiological stress on plaque instability in apolipoprotein E knockout mice. J Physiol Biochem 67: 401, 2011. 21512836 10.1007/s13105-011-0090-6

[192] Zheng X, Wang Q, Zhang Y, Yang D, Li D, Tang B, Li X, Yang Y, and Ma S. Intermittent cold stress enhances features of atherosclerotic plaque instability in apolipoprotein E-deficient mice. Mol Med Rep 10: 1679–1684, 2014. 25109747 10.3892/mmr.2014.2464

[193] Zhou H, Mehta S, Srivastava SP, Grabinska K, Zhang X, Wong C, Hedayat A, Perrotta P, Fernández-Hernando C, Sessa WC, and Goodwin JE. Endothelial cell–glucocorticoid receptor interactions and regulation of Wnt signaling. JCI Insight 5: e131384, 2020. 10.1172/jci.insight.131384 PMC7098785 32051336

[194] Zhou X, Robertson A-KL, Rudling M, Parini P, and Hansson GK. Lesion development and response to immunization reveal a complex role for CD4 in atherosclerosis. Circ Res 96: 427–434, 2005. 15662027 10.1161/01.RES.0000156889.22364.f1

